# Molecular and Functional Analysis of Sunitinib-Resistance Induction in Human Renal Cell Carcinoma Cells

**DOI:** 10.3390/ijms22126467

**Published:** 2021-06-16

**Authors:** Magdalena Rausch, Adriano Rutz, Pierre-Marie Allard, Céline Delucinge-Vivier, Mylène Docquier, Olivier Dormond, Jean-Luc Wolfender, Patrycja Nowak-Sliwinska

**Affiliations:** 1 School of Pharmaceutical Sciences, University of Geneva, CMU-Rue Michel-Servet 1, CH-1211 Geneva, Switzerland; Magdalena.Rausch@unige.ch (M.R.); Adriano.Rutz@unige.ch (A.R.); pierre-marie.allard@unige.ch (P.-M.A.); jean-luc.wolfender@unige.ch (J.-L.W.); 2 Institute of Pharmaceutical Sciences of Western Switzerland, University of Geneva, CMU-Rue Michel-Servet 1, CH-1211 Geneva, Switzerland; 3 Translational Research Center in Oncohaematology, 1205 Geneva, Switzerland; 4 iGE3 Genomics Platform, University of Geneva, 1206 Geneva, Switzerland; Celine.Delucinge@unige.ch (C.D.-V.); Mylene.Docquier@unige.ch (M.D.); 5 Department of Genetics and Evolution, University of Geneva, 1205 Geneva, Switzerland; 6 Department of Visceral Surgery, Lausanne University Hospital and University of Lausanne, 1015 Lausanne, Switzerland; olivier.dormond@chuv.ch

**Keywords:** acquired drug resistance, (clear cell) renal cell carcinoma, drug combination, isomerization, metabolites, sunitinib

## Abstract

Resistance in clear cell renal cell carcinoma (ccRCC) against sunitinib is a multifaceted process encompassing numerous molecular aberrations. This induces clinical complications, reducing the treatment success. Understanding these aberrations helps us to select an adapted treatment strategy that surpasses resistance mechanisms, reverting the treatment insensitivity. In this regard, we investigated the dominant mechanisms of resistance to sunitinib and validated an optimized multidrug combination to overcome this resistance. Human ccRCC cells were exposed to single or chronic treatment with sunitinib to obtain three resistant clones. Upon manifestation of sunitinib resistance, morphometric changes in the cells were observed. At the molecular level, the production of cell membrane and extracellular matrix components, chemotaxis, and cell cycle progression were dysregulated. Molecules enforcing the cell cycle progression, i.e., cyclin A, B1, and E, were upregulated. Mass spectrometry analysis revealed the intra- and extracellular presence of *N*-desethyl sunitinib, the active metabolite. Lysosomal sequestration of sunitinib was confirmed. After treatment with a synergistic optimized drug combination, the cell metabolic activity in Caki-1-sunitinib-resistant cells and 3D heterotypic co-cultures was reduced by >80%, remaining inactive in non-cancerous cells. These results demonstrate geno- and phenotypic changes in response to sunitinib treatment upon resistance induction. Mimicking resistance in the laboratory served as a platform to study drug responses.

## 1. Introduction

Through sustaining proliferative signaling, evading growth suppressors, and enabling replicative immortality, cancer cells may acquire resistance to anti-cancer drugs [1]. This is because a tumor may adapt to chronic drug administration and avoid medication-mediated growth control. Independent of the therapy type, i.e., chemotherapy, radiotherapy, and targeted therapies, the incidence of treatment resistance increases, making it more difficult to find and select the most beneficial treatment strategies [2,3,4,5]. Prevalent molecular mechanisms of resistance involve, e.g., genetic mutations, modifications up and downstream, and alternating the signaling transduction via compensatory pathways [6]. However, the detection of biomarkers and molecular drivers of resistance further guiding the treatment choice has not yet been fully adopted in the clinic.

Kidney cancer, especially clear cell renal cell carcinoma (ccRCC) accounts for one of the most difficult-to-treat cancers. Displaying an intrinsic or acquired treatment resistance determines treatment success [6]. Molecular drivers include mutated genes such as von Hippel-Lindau (*vhl*), intracellular and membrane-bound signaling proteins (growth factors and their receptors). Loss of function of the tumor suppressor gene *vhl* characterizes ccRCC and induces treatment resistance through consequent upregulation of the expression of hypoxia-inducible factor-1 (*hif-1*) genes. These genes code for the platelet-derived growth factor (PDGF) and vascular endothelial growth factor (VEGF), which stimulate angiogenesis and cell growth [4], contributing to the formation of a highly vascularized tumor environment. Enhanced cellular signal transduction via mitogen-activated protein kinases (MAPK) and the mechanistic target of rapamycin (mTOR) facilitate the sustained proliferation of the cancer cells.

Approaches to block these events promoting tumor growth and resistance development in ccRCC led to novel drugs. Small molecule-based targeted drugs, including kinase inhibitors with anti-cancer or anti-angiogenic activity, have emerged and are used as first-line treatment [7,8,9].

In 2006, the FDA approved the small molecule-based drug sunitinib (Sutent^®^). It is a multitargeted receptor tyrosine kinase inhibitor [10,11] and is frequently used as a first-line treatment for ccRCC. This drug predominantly targets the PDGF- and VEGF-receptors [12,13]. Nevertheless, resistance can be present intrinsically or can frequently develop after long-term treatment. In over 70% of patients initially responding to sunitinib, resistance develops within 15 months [14,15,16,17,18,19].

While the molecular mechanisms of acquired resistance to sunitinib have been unraveling rapidly, the mechanisms of intrinsic resistance remain elusive. The accurate definition of an acquired or intrinsic mechanism promoting resistance to drug treatment in cancer remains challenging as, differing from bacteria, no resistance-related genes or structures are expressed. Even upon the first treatment administration, an interplay between innate and adapted mechanisms will form. Acquired resistance to sunitinib presents itself through the accumulation of the drug in lysosomal vesicles [20], the presence of single nucleotide polymorphisms, the upregulated expression of proangiogenic growth factors, such as angiopoietin, HIF-1α/β, epidermal growth factor receptor (EGFR), fibroblast growth factor (FGF), VEGFR, as well as interleukins (IL-8), the adaptation of the tumor microenvironment [21,22,23], and almost twenty other mechanisms. Indeed, there is a consensus that the most critical drivers of sunitinib resistance are VHL, tyrosine-protein kinase Met (c-MET), VEGFR, and mTOR. These proteins are naturally dysregulated in ccRCC.

One of the essential characteristics in ccRCC is the strong association between cellular and drug metabolism [24,25]. Aberrations in metabolic pathways determine the treatment response, as well as the overall survival of patients [25,26,27]. Defective metabolism of drugs distorts pharmacokinetic and pharmacodynamics analysis in patient plasma. Inaccurate measures can also be a consequence of drug isomerism [28,29]. In the case of cisplatin, trans-isomerization impairs its clinical efficacy [30], while isomerization of cetirizine to levocetirizine [28] led to the development of a safer and more effective drug alternative.

Evaluating the anti-cancer efficacy, as well as the pharmacokinetics of sunitinib and its active metabolite (*N*-desethyl sunitinib), demonstrated high interpatient variability [31], potentially linked to the light-dependent isomerism of sunitinib [31,32]. The reversible isomerization from the (*Z*)- to the (*E*)-isomer occurs through light exposure, independent of the dose [31,32,33]. How isomerization and metabolism link to sunitinib resistance is not yet fully understood.

This study explored how resistance to sunitinib in human ccRCC cell lines can be established in the laboratory environment to induce molecular resistance mechanisms. We treated ccRCC cell lines for over 50 weeks repeatedly with sunitinib at increasing doses or with a steady dose of 1 µM, corresponding to clinically relevant sunitinib concentration. Because of the challenges to explain intrinsic resistance, in order to obtain a comparable cellular system, we treated cells with a single high-dose treatment (10 µM sunitinib), corresponding to doses measured in the tumor [20,34,35]. Therefore, we worked with two generated resistant clones and one cellular clone capable of surviving a high dose treatment, being insensitive (most likely intrinsically resistant) to sunitinib. Molecular changes of the chronically treated ccRCC cells were characterized by investigating the phenotypic alterations between sunitinib-naïve and sunitinib-resistant cells and treatment response after acquiring resistance. Using liquid chromatography coupled to high-resolution tandem mass spectrometry (LC-HRMS/MS), we detected the intra- and extracellular metabolites of sunitinib considered to be involved in sunitinib resistance.

Combination therapies, sunitinib re-challenge, and sequential therapy have been investigated to overcome resistance to sunitinib [2,36,37,38]. We further tested an optimized multidrug combination consisting of four tyrosine kinase inhibitors, AZD4547, osimertinib, AZD8055 and pictilisib [37], to reveal whether acquired resistance to sunitinib can be overcome.

## 2. Results

### 2.1. Establishment and Characterization of Acquired Resistance to Sunitinib in ccRCC Cell Lines

We developed sunitinib resistant (-SR) human ccRCC cell lines (Supplementary Figure S1A) by chronically treating Caki-1, A498, and 786-O cells [37] using increasing doses of sunitinib. We considered the cells as stably resistant once they became significantly insensitive to high dose (10 µM) treatment and when an accumulation of sunitinib in the cell body, more specifically in lysosomes, was confirmed (Supplementary Figure S1B–D). Due to the presence of auto fluorescence of sunitinib (excitation: 420 ± 20 nm, emission >470 nm) [21], we were able to image its localization without further counterstaining or manipulation of the compound. This was the case after 30 weeks of continuous treatment twice weekly during the passaging of the cells and was quantified through the increase in the effective dose reducing the viability of 50% of the cells (ED_50_; Supplementary Material Figure S1C). Furthermore, cells were maintained under chronic treatment with 1 µM sunitinib at each passage until experimental use.

**Figure 1:**
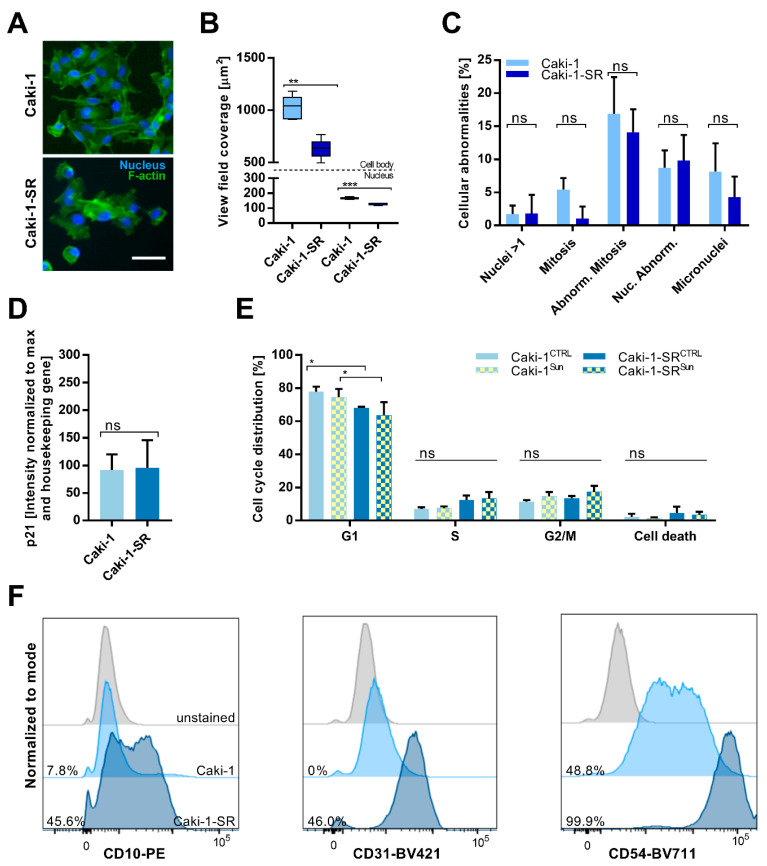
Identification of alterations between sunitinib-naïve Caki-1 and sunitinib-resistant Caki-1-SR cells. (**A**) Representative images of Caki-1 (top) and Caki-1-SR (bottom) cells fluorescently stained to visualize f-actin (green, GFP) and the nucleus (blue, Dapi). Scale bar = 20 μm. (**B**) Size of the cell body (above the dotted line) and the nuclei (below the dotted line) of Caki-1 and Caki-1-SR cells are expressed as the area covered in the view field. Error bars represent the SD. Statistical significance was calculated based on *n* = 3 independent experiments using Student’s *t*-test. ** *p* < 0.01, *** *p* < 0.001. (**C**) Bar graphs representing the appearance of cellular abnormalities analyzed in Caki-1 and Caki-1-SR cells. Error bars represent the SD. (**D**) Measurement of the expression of p21, a cyclin-dependent kinase inhibitor, in Caki-1 and Caki-1-SR cells. The protein expression was analyzed through Western blot experiments and is given as % intensity normalized to the maximal intensity measured as well as the housekeeping gene. (**E**) Cell cycle analysis demonstrating the number of cell given as % per cell cycle phase (G1, S, G2/M and cell death). Error bars represent the SD. Statistical significance was calculated based on *n* = 2–3 independent experiments using Student’s *t*-test and two-way ANOVA with unequal variances; * *p* < 0.05. (**F**) Histograms showing the differential expression of the surface proteins CD10 (left graph), CD31 (middle graph) and CD54 (right graph). The expression was detected using flow cytometry experimentation comparing the unstained control (grey), Caki-1 cells (light blue) and the Caki-1-SR cells (dark blue).

The morphology of the resistant cells varied from one of the sunitinib-naïve cells (Figure 1A and Supplementary Figure S2A), deforming the cell body and demonstrating changes at the nuclear level, i.e., the presence of multiple nuclei per cell. To determine the size of the nuclei and the cell body, we evaluated the area covered in the view field of the microscopic images (view field coverage–1224 × 904 pixels at 10× magnification). Interestingly, these results show that, comparing Caki-1 and Caki-1-SR cells, the size of the nuclei and the cell body are significantly smaller (Figure 1B). Similar results were obtained for A498-SR, whereas 786-O-SR cell body size was not modified significantly. The nuclear size was significantly reduced in 786-O-SR cells, in contrast to A498-SR cells, where the size of the nuclei was increased (Supplementary Figure S2B). The analysis of abnormalities related to the nuclei and mitosis demonstrated no significant alterations, either in A498-SR or Caki-1-SR or 786-O-SR cells (Figure 1C and Supplementary Figure S2D). Measurement of the expression of p21 revealed no p21 increase in Caki-1-SR cells compared to Caki-1 cells (Figure 1D), or in 786-O-SR cells (Supplementary Figure S2E, right graph). Caki-1-SR and 786-O-SR cells did not present a phenotype that would indicate a malfunction of mitotic events. However, in A498-SR cells, the expression of p21 was increased (231% vs. 61% Supplementary Figure S2E, left graph). These results suggest that a dormancy-like phenotype can be induced upon sunitinib treatment, likely dependent on the genetic background and susceptibility of the cells [39,40].

### 2.2. Sunitinib-Resistance in Cell Cycle Analysis and Cell Surface Protein Expression

In the next step, we analyzed the distribution of cells within the distinct phases of the cell cycle measured by flow cytometry. Significantly fewer cells were observed in the G1 phase upon treatment with 1 µM sunitinib (Figure 1E, Caki-1-SR^CTRL^ and Caki-1-SR^Sun^). We did not detect significant changes in the cell cycle in A498-SR and 786-O-SR cells (Supplementary Figure S2G). Connecting the data of phenotypic and mitotic modifications in response to chronic sunitinib treatment (>30 weeks), Caki-1-SR and 786-O-SR cells were able to adapt and resist the treatment, and thus were able to proliferate again without restraint, although 1.5-fold slower than the parental cells. However, A498-SR cells demonstrated aberrations related to delayed mitosis represented through an increased nuclear size, upregulated expression of p21 in the absence of a G1 and G2/M blockade.

Characterization of the expression of cell surface proteins revealed the upregulation of CD10 (cell membrane metallopeptidase), CD31 (platelet endothelial cell adhesion molecule, PECAM-1) and CD54 (intercellular adhesion molecule 1, ICAM1) in Caki-1-SR cells (Figure 1F and Supplementary Figure S3) compared to Caki-1 cells. The selection of these markers was based on patient histology and pre-characterization of the cells through flow cytometry to define the expression patterns [41,42,43,44]. Further characterization of the expression of PD-L1 on sunitinib-naïve and sunitinib-resistant ccRCC cell lines revealed that 786-O and 786-O-SR cells present a high density of PD-L1 on their cell surface. Remarkably, the chronic treatment with sunitinib reduced the expression of PD-L1 on A498-SR and Caki-1-SR cells compared to the sunitinib-naïve cells (Supplementary Figure S4).

### 2.3. Modifications in Protein and Gene Expression upon Sunitinib Resistance Induction

**Figure 2:**
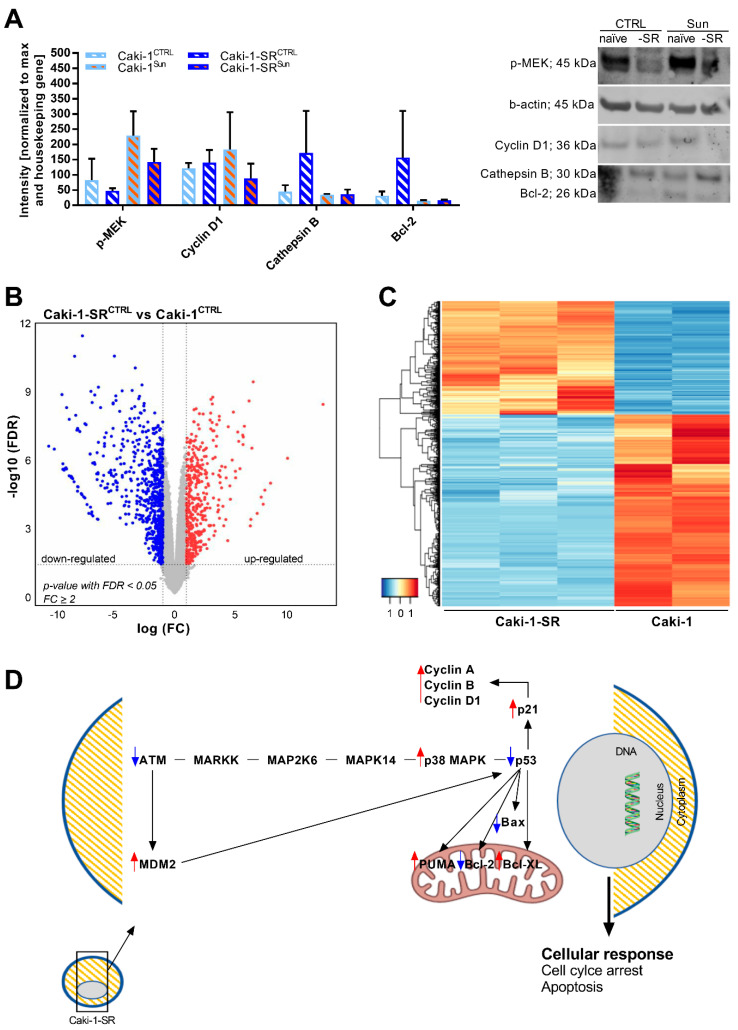
Analysis of the gene and protein expression of Caki-1 vs. Caki-1-SR cells. (**A**) Expression of proteins (*n* = 2) related to cell signaling (phosphorylated MEK; p-MEK), mitosis (cyclin D1), proteolysis (cathepsin B), and apoptosis (Bcl-2). The level of expression is presented as the intensity of the bands on nitrocellulose membrane after Western blot analysis normalized to the maximal intensity and the housekeeping gene β-actin. Error bars represent the SD. (**B**) Volcano plot showing 12,940 genes, with each dot representing a gene. The red dots are significantly upregulated genes in Caki-1-SR^CTRL^ compared to the Caki-1^CTRL^, while the blue dots down-regulated genes (thresholds: *p*-value with false discovery rate (FDR) < 0.05 & FC ≥ 2). (**C**) Heat map of the 1050 significant differentially expressed genes (thresholds: *p*-value with FDR) < 0.05 and FC ≥ 2 genes comparing Caki-1 (*n* = 2) and Caki-1-SR cells (*n* = 3). Genes that are upregulated in their expression are shown in red, while genes that are downregulated are shown in blue. For more detailed information, see Supplementary Tables S1 and S2. (**D**) Demonstration of the most dominant altered genes in Caki-1-SR cells inducing a cell cycle arrest but prohibiting apoptosis. Legend: ATM = serine/threonine kinase; MARKK, MAP2K6, MAPK14, p38 MAPK = protein kinase; p53 = tumor suppressor protein, p21 = cyclin-dependent kinase inhibitor; Bax = Bcl2-associated X protein; Bcl-2 = B-cell lymphoma 2 protein; PUMA = p53 upregulated modulator of apoptosis.

Selecting Caki-1 and Caki-1-SR, originated from an atypic skin metastasis of ccRCC, as representative ccRCC cell line for all further experiments, we first evaluated the protein expression upon sunitinib resistance induction performing Western blot experiments (Figure 2A and Supplementary Figure S5). In general, measuring the expression level of phospho-mitogen-activated protein kinase (p-MEK) in Caki-1 and Caki-1-SR cells (CTRL) revealed that Caki-1-SR cells expressed 1.7-fold less p-MEK (82.3 vs. 47.8). These results indicate that signaling via p-MEK was downregulated in Caki-1-SR cells. Upon treatment with 1 µM sunitinib for 72 h, the expression of p-MEK increased in Caki-1 cells 2.9-fold (82.3 vs. 228.9) and Caki-1-SR cells 3-fold (47.8 vs. 143.0). Similarly, the expression of cyclin D1, a protein promoting the cell cycle G1/S transition, was increased 1.5-fold in Caki-1 cells while it was decreased 1.6-fold in Caki-1-SR cells. This is in agreement with our flow cytometry analysis (Figure 1E). The expression of cathepsin B and Bcl-2, proteins involved in apoptosis, was increased 3.8- and 4.9-fold (cathepsin B: 171.7; Bcl-2: 156.9) in the Caki-1-SR^CTRL^, respectively, but remained at an equal level in the other conditions (cathepsin B < 45; Bcl-2 < 32). All data were non-significant.

**Figure 3:**
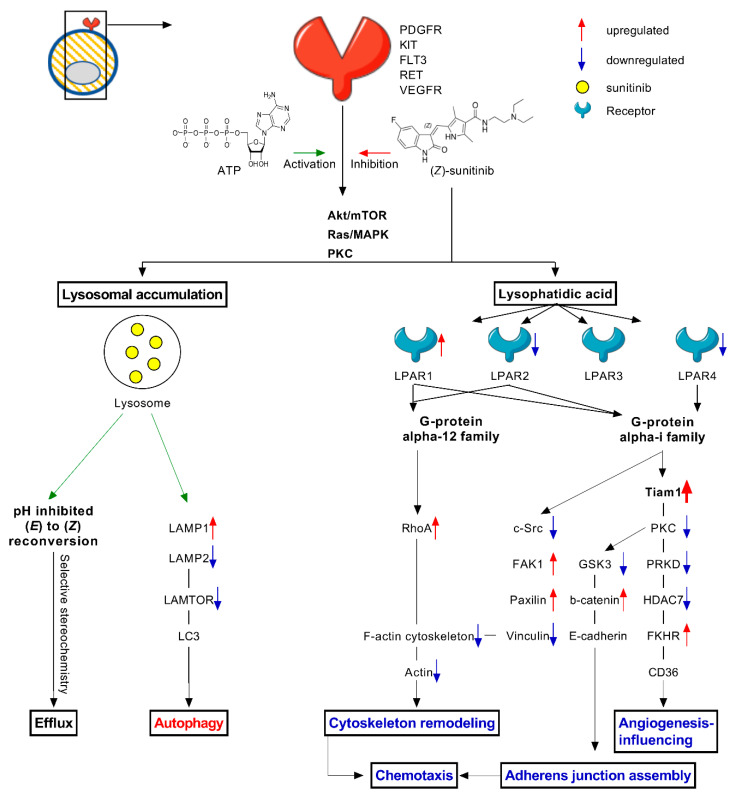
Cellular changes induced through chronic treatment with sunitinib resulting in sunitinib resistance. Schematic representation of induced cellular changes in response to sunitinib treatment, mainly focusing on autophagy, chemotaxis and stereoisomerisation after lysosomal accumulation. Sunitinib inhibits the signal transduction via Akt/mTOR, Ras/MAPK and PKC through binding to the ATP-binding site of numerous cell membrane receptors, i.e., PDGFR, c-KIT, FLT-3, RET, VEGFR. Lysosomal accumulation: Lysosomes store sunitinib, where the acidic pH inhibits the reconversion from (*E*) to (*Z*). Cells might have intrinsic selectivity to stereoisomers, which could be linked to efflux. Lysosomal storage further induces autophagy by dysregulating autophagy-related proteins, i.e., LAMP, LAMTOR, MAPK and MTOR activator and LC3. Lysophatidic acid: Sunitinib treatment induces the production of lysophatidic acid, which binds to lysophosphatidic acid receptors (LAPR) that are linked to G-protein signaling. Dependent on the subsequent signaling pathway, cellular functions, i.e., cytoskeleton remodeling, adherens junction assembly, angiogenesis-influencing proteins and chemotaxis, will be downregulated. Legend: ATP = adenosine triphosphate; CD36 = integral membrane protein; c-Src = protein kinase; FAK = focal adhesion kinase; FKHR = Forkhead box protein O1 (FOXO1); FLT3 = fms like tyrosine kinase 3; GSK3 = serine/threonine protein kinase (Glycogen synthase kinase 3); HDAC = histone deacetylase; KIT = tyrosine kinase; LAMP = lysosome associated membrane protein; LAMTOR = endosomal/lysosomal adaptor and mitogen activated protein kinase; LAPR = lysophosphatidic acid receptor; MAPK = mitogen activated protein kinase; PDGFR = platelet derived growth factor receptor; LC3 = light chain 3; PKC = protein kinase C; PRKD = serine/threonine protein kinase; RET = tyrosine protein kinase receptor; RhoA = Ras homolog family member A; Tiam = T-lymphoma invasion and metastasis-inducing protein 1; VEGFR = vascular endothelial GFR.

To characterize global transcriptome and molecular changes in response to chronic sunitinib treatment, we performed RNA sequencing comparing Caki-1 and Caki-1-SR cells. The analysis demonstrated differential gene expression on 1193 genes (adj. *p*-value <0.05). This accounts for 37% of upregulated and 63% downregulated transcripts. Pathway analysis revealed that the affected transcripts belong to various signaling pathways and cellular functionalities, i.e., lysosomal chemotaxis (Figure 3 and Supplementary Table S1), positive regulation of Wnt/β-catenin signaling (Supplementary Figure S6), DNA damage control at the stage of G2/M checkpoint control, and activation of p53 (Supplementary Figure S7). Following GeneOntology and Kyoto Encyclopedia of Genes and Genomes database analysis, we were able to cluster another 46 significantly dysregulated genes in the calcium signaling pathway, as well as in the interaction process of cytokine-cytokine receptors (Figure 2B–C). Highlighting the signaling pathways regulating DNA damage control and p53 activation demonstrated that molecules promoting the cell cycle progression were upregulated, i.e., p38 MAPK, cyclins. In contrast, molecules inducing apoptosis were downregulated, i.e., p53, Bax, Bcl-2 (Figure 2D). We further elucidated that various adhesion molecules became dysregulated (Figure 3 and Supplementary Table S2).

The RNA sequencing data showed changes in the RNA levels of VHL, c-MET, VEGF, mTOR and PDGF(R) (Supplementary Table S3), but they were not significant. These proteins are used as biomarkers for the detection of ccRCC and are targets of sunitinib.

Additional dominant cellular changes after resistance to sunitinib was acquired by Caki-1-SR cells are demonstrated in Figure 3. The data revealed that resistance to sunitinib is related to lysosomal storage, (i) potentially altering the lysosome-mediated drug efflux and (ii) activating autophagy. The production of lysophatidic acid modified the signal transduction via lysophosphatidic acid receptors (LPAR) coupled to G-protein signaling. Determined by the activated signaling pathway, various cellular functions were downregulated; as a consequence of the upregulation of the small GTPase protein RhoA, the cellular capacity to remodel the cytoskeleton was decreased. Downregulation of the proto-oncogene tyrosine-protein kinase Src, the serine/threonine-protein kinase GSK3 and protein kinase C (PKC) diminished cellular adhesion, chemotaxis, and angiogenesis. The formation of new vessels, called angiogenesis, is not performed but promoted by cancer cells. After chronic sunitinib treatment, genes were downregulated, participating in the production of angiogenesis-promoting stimuli. The anti-angiogenic effects of targeted small molecule-based drugs, i.e., sunitinib and axitinib, are mediated by the direct blockade of VEGFR and PDGFR expressed on cancer and especially on endothelial cells [35]. Even if the most potent activity is an anti-angiogenic one on the endothelial cells, it affects the cancer cells by targeting exactly those genes participating in enhancing angiogenesis in the tumour.

### 2.4. Stereoisomers of Sunitinib and Metabolites in Caki-1-SR Cells

**Figure 4:**
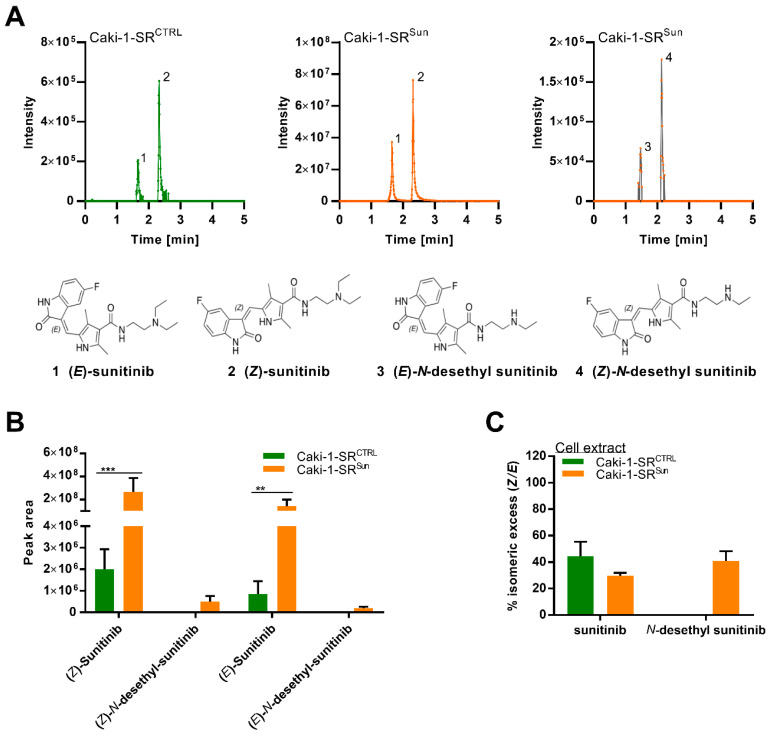
Liquid chromatography-high resolution tandem mass spectrometry analysis of sunitinib-related compounds in Caki-1-SR cells. (**A**) LC-HRMS/MS analysis (base peak intensity chromatograms) of the cell extract of Caki-1-SR in the absence (Caki-1-SR^CTRL^) and presence of 1 µM sunitinib (Caki-1-SR^Sun^) to demonstrate the presence of the (*E*)- and (*Z*)-stereoisomers of sunitinib and its *N*-desethyl metabolites. (**B**) Peak area of sunitinib and (N)-desethyl sunitinib isomers in the extract of Caki-1-SR^CTRL^ and Caki-1-SR^Sun^ (*n* = 3). Error bars represent the SD. Statistical significance was calculated by using a two-way ANOVA with unequal variances; ** *p* < 0.01, *** *p* < 0.001. (**C**) Bar graphs demonstrating the % isomeric excess (*Z/E*) of the sunitinib isomers and their *N*-desethyl metabolites in cell extract (*n* = 3) of Caki-1-SR cells cultured for 24 h in culture medium (CTRL) or in culture medium supplemented with 1 μM sunitinib.

Investigation of the conversion of sunitinib in Caki-1-SR cells through LC-HRMS/MS was performed and showed two peaks corresponding to its (*E*) and (*Z*)-isomer (*m/z* = 399.2183; Figure 4A and Supplementary Figure S8A). Diverse studies showed the presence of both stereoisomers, (*E*)-sunitinib and (*Z*)-sunitinib [23,31,33]. Attribution was made by comparing the retention times and stereoisomeric ratios with the literature [32] and a standard solution in methanol (Supplementary Figure S8A). (*E*)-sunitinib (Figure 4A, 1) appears to be clinically inactive, and its isomerization occurs through light exposure [31,33]. (*Z*)-sunitinib (Figure 4A, 2) is clinically relevant and active. The two stereoisomers could be detected at the same ratios in the supernatant, independent of the dose of sunitinib, with the percent of stereoisomeric excess (see Materials and Methods) being approximatively 85% (*Z/E*) at all concentrations (Supplementary Figure S8B), which is slightly lower than the values reported in the literature [31,33]. The increasing percentage of stereoisomeric excess at low concentrations can be attributed to the limit of detection of peak 1 (see Materials and Methods).

When comparing the supernatant and the cellular extract, remarkable differences in the ratios of (*Z*)-sunitinib and its stereoisomer were observed. While stereoisomeric excess of the (*Z*)-form was observed (>96%) in both Caki-1-SR^CTRL^ and supernatants of sunitinib-treated Caki-1-SR cells (Caki-1-SR^Sun^) (Supplementary Figure S8) [31,33], in the cell extract, the stereoisomeric excess decreased to 45% and 30%, respectively. The presence of (*Z*)-*N*-desethyl sunitinib and (*E*)-*N*-desethyl sunitinib in the cell extract of Caki-1-SR cells cultured for 24 h in the culture medium treated with 1 µM sunitinib was detected, whereas, in the supernatant, only the (*Z*)-form could be detected (Figure 4B). After 24 h of treatment, the concentration of sunitinib and its *N*-desethyl metabolite was higher in the cell extract than in the supernatant (Supplementary Figure S8C).

### 2.5. Combination Treatment Overcomes Resistance in Different Sunitinib-Resistant Caki-1 Clones

**Figure 5:**
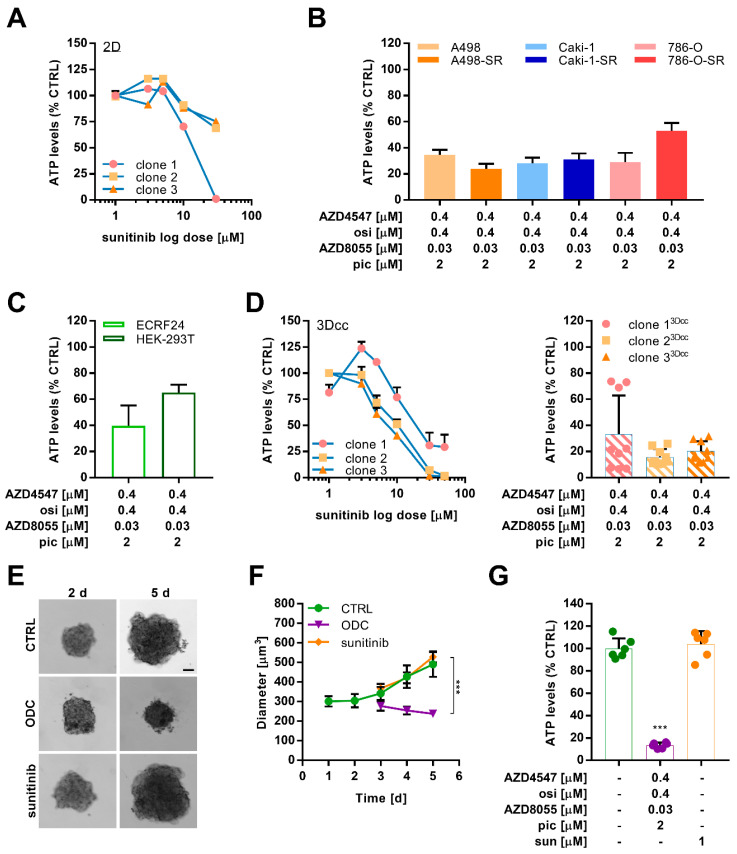
Tumor growth of Caki-1 and Caki-1-SR in vivo and ex vivo use for testing of optimized drug combinations. (**A**) Viability, measured as ATP levels, of sunitinib-resistant clones in a 2D monolayer (2D). (**B**) Response of sunitinib-naïve and -resistant ccRCC cells to an optimized multidrug combination treatment (ODC) containing AZD4547 (0.4 µM), osimertinib (osi, 0.4 µM), AZD8055 (0.03 µM), and pictilisib (pic, 2 µM). (**C)** Effect of the ODC on non-cancerous cells ECRF24 and HEK-293T. (**D**) Viability, measured as ATP levels, of sunitinib-resistant clones in heterotypic 3D co-culture (3Dcc) with 10% endothelial cells (ECRF24) and 20% fibroblasts (NHDFα cells) in response to increasing doses to sunitinib (left graph) and the ODC (right graph). Error bars represent the SD. (**E**) Representative bright-field images of murine ex vivo spheroids taken on days 2 and 5 (2–5 d) after seeding. ODC treatment was applied onto the spheroids on 2 d and maintained for 72 h until 5 d.). Sunitinib was applied at a concentration of 1 µM. Scale bar = 100 µm. (**F**) Size measurements of the diameter of the murine ex vivo spheroids in time. Spheroids remained untreated (CTRL) or were treated with either the ODC or 1 µM sunitinib. Error bars represent the SD of six spheroids per condition (*n* = 6). (**G**) Bar graphs representing the ATP levels (viability) as % compared to the CTRL in response to 72 h treatment with ODC and sunitinib. Error bars represent the SD of six spheroids per condition (*n* = 6). Statistical significance was calculated based on *n* = 6 independent experiments by using a one-way ANOVA with unequal variances; *** *p* < 0.001.

We used the three distinct sunitinib-resistant Caki-1 clones (Supplementary Figure S1) to explore the insensitivity to sunitinib in 2D (Figure 5A). Measuring the ATP levels as a reflection of cell viability, all clones were insensitive to treatment with sunitinib at doses between 1 and 10 µM. At a dose of 30 µM, the ATP levels of clone 1 were decreased by >95%, whereas the ATP levels of clone 2 and 3 indicated that >70% of the cells were able to resist the high dose of 30 µM.

In parallel, we evaluated the activity of an optimized low-dose synergistic drug combination (ODC), previously optimized by us in Caki-1-SR cells [37]. This drug combination was developed using the validated phenotypic approach called Therapeutically Guided Multidrug Optimization [37,38,45,46] and consisted of four tyrosine kinase inhibitors [37], namely AZD4547, osimertinib, AZD8055, and pictilisib (Supplementary Table S4). This ODC was optimized to selectively target Caki-1-SR clone 1. We cross-validated the activity of this ODC in all ccRCC cells (Figure 5B) and in non-cancerous cells ECRF24 (endothelial cells) and HEK-293T (human-embryonic kidney, Figure 5C). The results demonstrate that the ODC reduced the ATP levels of all ccRCC cells by 66.7% on average. Simultaneously, the ODC showed anti-angiogenic activity, reducing the ATP levels of ECRF24 cells by 60.3%. In contrast, HEK-293T cells were not targeted by the treatment.

In the next step, we used the sunitinib-resistant Caki-1 clones to establish the heterotypic 3D co-cultures (3Dcc), including human endothelial cells (ECRF24) and human fibroblasts (NHDFα) closely mimicking the physiologic characteristics of ccRCC (Figure 5D, left graph, Supplementary Figure S9) [37,47,48].

Interestingly, 3Dcc based on clones 2 and 3 were more sensitive to increasing doses of sunitinib than clone 1, which was the opposite in monolayer conditions. These results further indicate that one treatment with a high dose of sunitinib (50 µM) harmed all three 3Dcc, strongly decreasing the ATP levels by >70% (Figure 5D, left graph). These data highlighted that 3Dcc was similarly affected by sunitinib treatment than the cancer cells cultured solely in 2D. The same culture model was used to evaluate the efficacy of the ODC and interestingly, all three clones cultured in 3Dcc were similarly affected by the ODC treatment, reducing the ATP levels significantly (Figure 5D, right graph).

### 2.6. Optimized Multidrug Combinations overcome Sunitinib Resistance in Ex Vivo Organoid-Like Cultures of Caki-1-SR Organoids

We inoculated the Caki-1 and Caki-1-SR clone 1 subcutaneously in male and female Swiss nu/nu mice (Supplementary Figure S10A) to evaluate the capacity to form tumors in vivo. Tumors were allowed to develop, and the kinetic growth was comparable in time (Supplementary Figure S10B). Mice with Caki-1-SR clone 1 tumors received treatment with 20 mg/kg sunitinib, a dose known to reduce the tumor growth of Caki-1-based tumors by approx. 50% [49] and that was inactive in Caki-1-SR-based tumors (Supplementary Figure S10B).

In the next step, we used dissected Caki-1-SR tumor tissues to obtain organoid-like cultures after tissue dissociation. We did not isolate distinct cell populations but maintained all cell cohorts present in the subcutaneously grown tumor. By preserving all the different cell types, we were able to form organoid-like cultures with a similar appearance as in vitro 3D co-cultures. The organoid-like cultures were maintained for 2 days and afterward re-dissociated to obtain a single cell suspension to prepare more homogeneous organoid-like structures (Figure 5E and Supplementary Figure S10C) that were used for optimized drug combination validation. Incubation of organoids with ODC treatment for 72 h reduced the growth of the organoids significantly compared to the positive control (1 µM sunitinib), the sham-control (culture medium supplemented with 0.05% DMSO) and the single drug treatments (Figure 5F and Supplementary Figure S10D). We analyzed the viability of the organoid-like cultures through the ATP level measurements, revealing the anti-cancer activity of ODC (ATP level reduction by 90%; Figure 5G) and its monotherapies (Supplementary Figure S10E).

## 3. Discussion

In this study, we evaluated the consequences of sunitinib resistance induction in human RCC cell lines at molecular, morphometric, and functional levels. Our findings align with resistance mechanisms described in vitro and in patient tissue [2,14,15,22,50,51,52,53]. The production of cell membrane and extracellular matrix components, chemotaxis, and cell cycle progression were dysregulated compared to sunitinib-naïve cells.

Sunitinib resistance induction led to morphometric changes in RCC cells, such as alterations in the cellular and nuclear size (Figure 1 and Supplementary Figure S2). Our group [37] and others have previously reported on apparent morphometric changes in treatment-resistant cells, i.e., heterotrophy [52,54,55], enhanced cell–cell interactions through an increased number of short- and long-distance cell–cell contacts profoundly stabilizing focal adhesions [56], or the formation of tunneling nanotubes [57,58]. These morphometric changes were accompanied by deformations of the actin cytoskeleton de-shaping the cell body. In this study, we show that over 70 genes related to cell adhesion were dysregulated significantly upon chronic sunitinib treatment. In particular, anchoring proteins, i.e., collagen, fibronectin, and laminin, connecting cells with the extracellular matrix or the microenvironment were downregulated (Figure 2 and Supplementary Table S2). The downregulation of extracellular matrix receptors and cell adhesion pathways were reported by Li et al.by proteomic analysis after 4 years of treatment with sunitinib [59]. Furthermore, the production of cell plasma components, signaling, and lysosomal function appeared to be affected (Figure 3).

The upregulation of CD10, CD31 and CD54 after sunitinib treatment with especially CD31 and CD54 participating in cell–cell interactions and cell attachment. CD10, also known as neprilysin, is a zinc-dependent metalloprotease that cleaves peptides and has shown to be associated with treatment resistance in head and neck squamous cell carcinoma [42,60].

Sequestration of sunitinib in lysosomes is one of the known sunitinib resistance mechanisms (Supplementary Figure S1) [20,21,23]. Lysosomal accumulation occurs mainly during the administration of low concentrations of sunitinib [20,23]. The release of sunitinib from the lysosomes can be induced through light exposure, re-activating the anti-cancer efficacy [21]. The photoactivated release was obtained through irradiation (λ = 420 nm) with two different doses (34 and 130 J/cm^2^) [21], whereas isomerization occurs through exposure to white light [31]. We measured changes in lysosomal trafficking through RNA sequencing and alterations in the expression of the lysosomal protease CTSB (cathepsin B; Figure 2). It has been shown that the downregulation of cathepsin B led to incomplete autophagy by inducing the formation of autolysosomes [23]. The role of autolysosome formation and the stability of lysosomes in response to sunitinib treatment have further been demonstrated in pancreatic cancers. Upon treatment with sunitinib, pancreatic cancer cell lines increased autophagy in vitro, presented through the upregulation of LC3B-II levels [61]. It has been also shown that the inhibition of autophagy, by downregulating the expression of lysosome-associated membrane protein (LAMP2; Figure 3 and Supplementary Table S1), simultaneously reduces the expression of autophagy-related protein 5 and 7, enhancing the treatment efficacy of sunitinib [34,61]. Modifications of autophagy are often linked to modifications in mitosis and apoptosis.

Flow cytometry analysis did not reveal significant changes when comparing the cell cycle distribution of sunitinib-naïve and sunitinib-resistant cells, indicating that resistant cells can continue to proliferate by progressing mitosis. Connecting this result with RNA sequencing data showed the upregulation of mitosis promoting proteins (p21, cyclins; Figure 2) and simultaneous downregulation of apoptosis-inducing proteins (p53, Bax, Bcl-2; Figure 2). As resistance to sunitinib is multifaceted and depends on the mode of application, alterations in mitotic processes can vary, involving G2/M arrest or G1 blockade [23,52]. Independent of the drug administration and the occurrence of these events, resistant cells manage to snap out of these impediments and start to proliferate unrestrainedly.

Sunitinib and its active metabolite (*N*-desethyl sunitinib) are prone to spontaneous isomerization induced through light exposure. Two stereoisomeric forms are known and have been detected in our study (Figure 4), the *E* (trans) and *Z* (cis) isomer [31,32,33]. The isomerization limits the accurate quantification of both stereoisomeric forms in cellular extracts or the plasma of patients. It has been shown that light-induced isomerization can be reversed through pH, heat, and light protection. The concentration of sunitinib did not appear to impact the process of (re-)isomerization [32]. Using liquid chromatography coupled to high-resolution tandem mass spectrometry (LC-HRMS/MS), we detected both stereoisomers as well as their metabolites in Caki-1-SR cells. Sato et al. postulated that sunitinib resistance in RCC could be analyzed by monitoring changes in metabolites, leading to the identification of new therapeutic targets [33]. Our results indicate that Caki-1-SR cells first accumulate sunitinib and then ‘secrete’ it into the supernatant, independently of the treatment. The percent of stereoisomeric excess of the (*Z*)-form was 2-fold lower in the cell extracts than in the supernatant, thus suggesting that cells store the (E)-form preferentially. Stereoselective compound stability might be due to fine compartment-specific milieus, i.e., acidic lysosomes and neutral medium. Currently, there is no explanation of the biological processing of the (*Z*)- versus the (*E*)-form, but we are confident that based on our findings, continued research will assist in understanding resistance to sunitinib and explain stereoselectivity. We assume that cellular ‘self-protection’ mechanisms depend on the accumulation and secretion of sunitinib, as well as the reconversion inhibition through acidic pH in lysosomes. During re-challenge with 1 µM sunitinib, the metabolic conversion of sunitinib to *N*-desethyl sunitinib occurred. Further studies are needed to elucidate the stereospecific storage, metabolism, and secretion processes of sunitinib in sunitinib-resistant cells. After sunitinib was administered for 24 h, a higher concentration of the parental compound and the metabolite were measured in the cell extract in confront to the supernatant (Supplementary Figure S8). If sunitinib uptake, storage and metabolism are certain, efflux remains to be evaluated. Dependent on intra- and extracellular efflux, compound inter-and extracellular ratios of the compounds might change and long-term experiments need to be conducted to investigate these mechanisms.

Establishing individual sunitinib-resistant clones served in the development of a multifaceted cellular platform, which represents intrinsic and acquired resistance towards sunitinib in RCC. Using adapted protocols to facilitate the evolution of resistance led to the understanding that the sensitivity to treatment can be dependent or independent of the geno-/ phenotype. Our results demonstrate that 3D co-cultures were more sensitive to increasing doses of sunitinib (Figure 5), which can be a cause of altered cell signaling or interactions with other cells in the format of a heterotypic 3D co-culture. This is in agreement with studies reporting a dominant modification in receptor tyrosine kinase signaling via the Akt-mTOR pathway in homotypic 3D cultures modeling colon and breast cancer [62,63,64,65]. Depending on the culture technique and the support through a scaffold or specified condition medium, the response to drug regimens may vary by culturing RCC-based homotypic 3D co-cultures in a stem cell medium [66].

The validation of an optimized drug combination consisting of tyrosine kinase inhibitors [37] showed that targeting Caki-1-SR cells had a potent anti-cancer activity and was able to overcome sunitinib resistance mechanisms. The blockade of the mitogen-activated kinase signaling pathway at various levels of the signaling cascade significantly reduced the viability of the Caki-1-SR clones and ex vivo cultured Caki-1-SR cells. Our previous study demonstrated the roust anti-cancer efficacy of seven different TKI-based ODCs in three sunitinib-naïve, three sunitinib-resistant ccRCC cell lines, and the anti-angiogenic efficacy in one endothelial cell line [37]. Analyzing the TK activity in RCC cell line and patient samples, Haake et al. revealed numerous unique tyrosine phosphosites in EGFR, MET, JAK2, and FAK [67], highlighting the strong potential for therapeutic targeting. Furthermore, TKI resistance can be induced through the dominant signaling via VEGFR, other TKs, or downstream pathways. Therefore, the multi-target blockade through combination therapies became promising strategies for RCC treatment [68]. Our data revealed the importance of inhibiting extra- and intracellular targets of TK signaling [69,70,71,72,73,74,75].

In summary, we demonstrated that sunitinib resistance was represented accurately in vitro in human ccRCC cell lines and was overcome through the treatment with an optimized drug combination targeting TK signaling. Molecular changes acquired through resistance induction were characterized by evaluating phenotypic and mitotic alterations. Through LC-HRMS/MS, we detected sunitinib metabolites involved in sunitinib resistance in both intra- and extracellular extracts. Our results indicate that sunitinib induction can be reliably induced in laboratory settings, and those sunitinib-resistant cells may serve as a platform to study drug responses.

## 4. Materials and Methods

### 4.1. Cells

A498, Caki-1 and 786-O, human renal cell carcinoma cell lines, were purchased from ATCC. All cells were cultured in a humidified incubator with 5% CO_2_ at 37 °C. A498 and Caki-1 cells were kept in DMEM medium (Thermofisher, Basel, Switzerland, Gibco, 31966021), 786-O in RPMI medium (Gibco, 61870010).

### 4.2. Drugs

Sunitinib was kindly donated by the University Hospital of Geneva, dissolved in sterile DMSO (Sigma-Aldrich, Buchs, Switzerland, D8418-50ML), and diluted in a culture medium. Compounds used in the ODC were purchased, dissolved, and stored as previously described [37]. As all compounds were dissolved in DMSO and upon dilution, a concentration of 0.05% was present in the treated conditions, and we used 0.05% DMSO in culture medium as sham-control (CTRL). This sham-control was used to normalize the treatment results referred to as % CTRL.

### 4.3. Fluorescence staining and cell cycle distribution

Cells were fixed, permeabilized, and stained on 24-well plate glass inserts, as previously described [37]. Cells were double-stained, applying 1:200 dilution of Alexa Fluor 488 conjugated phalloidin (Invitrogen, Carlsbad, CA, USA, A12379) and afterwards 1:2500 dilution of Dapi. Images were taken with a Biotek Citation 3 (BioTek Instruments, Sursee, Switzerland) with corresponding software at the default settings. Pictures were analyzed with CellProfilerTM and Adobe^®^ Photoshop.

Cells were stained with FxCycleTM PI/RNase staining solution (Invitrogen, F10797) to analyze the cycle distribution on an AttuneTM NxT flow cytometer (Thermofisher) with corresponding software.

### 4.4. Fluorescence-Activated Cell Sorting

A total of 1 × 10^5^ to 5 × 10^5^ cells were harvested for fluorescence-activated cell sorting (FACS) analysis. Cells were washed twice with PBS before the addition of antigen-specific anti-human monoclonal antibodies (Supplementary Table S5). Analysis was performed on a Beckton Dickinson (BD)LSRFortessa (5 lasers; Franklin Lakes, NJ, USA) and BD FACSDivaTM software.

### 4.5. ATP Level Measurements

CellTiter-Glo solution (Promega, Dübendorf, Switzerland, G7572) following the product instructions was used to analyze the ATP using a luminescence-based read-out. The luminescence read-out was performed on a Biotek Citation 3 (BioTek instruments) with corresponding software (Gen5, version 3.04) at the default settings.

### 4.6. Three-Dimensional Heterotypic Spheroid Cultures from Cell Lines

Scaffold-free heterotypic spheroidal cultures of 700 ccRCC cells, 200 NHDFα, and 100 ECRF24 cells were prepared in 96-well low attachment U-bottom plates (GreinerBio, 650970) All bright field and fluorescence images were obtained using a Biotek Citation 3 (BioTek instruments) with corresponding software (Gen5, version 3.04) with the default settings.

### 4.7. Western Blot

A total of 1 × 10^6^ single cells (2D) were harvested, washed twice with ice-cold PBS, and lysed in 1× RIPA buffer containing protease inhibitor cocktail (Roche, Basel, Switzerland) and PhosSTOP (Roche, Basel, Switzerland). Protein concentrations were quantified with Bradford assay (Thermo Fischer Scientific, Waltham, MA, USA). Fifty micrograms of whole protein for each condition were loaded on 4%–12% polyacrylamide gels (Invitrogen, Waltham, MA, USA), separated at 120 V for 1.5 h, and blotted at 25 V for 2 h via wet-transfer onto nitrocellulose membranes. Membranes were blocked for 1 h at RT using Odyssey blocking buffer (LI-COR Biosciences, Lincoln, NE, USA) before overnight incubation with primary antibodies (Supplementary Table S6). After four washing steps, membranes were incubated with secondary antibodies for 1 h at RT. Bands from immunoreactive proteins were visualized by an Odyssey infrared imaging system at 700 nm for α-mouse and 800 nm for α-rabbit-stained proteins. Analysis was performed using Image Studio^TM^ Lite software.

### 4.8. RNA Sequencing

Total RNA content was isolated using the RNA easy^®^ Plus Kit (74134, Qiagen, Hilden, Germany) following the manufacturer’s instructions. The RNA quality control was done together with library preparation using TruSeqHT Stranded mRNA (Illumina). RNA samples were sequenced on an Illumina HiSeq 4000 System using 100-bp single-end reads protocol. Quality control was done with FastQC v.0.11.5. All reads were outlined to the human genome (UCSC hg38) using STAR v.2.5.3a software [76] with an average alignment at 92%. PicardTools v.2.9.0 was used to incorporate biological quality control. HTSeq v.0.9.1 was used to obtain raw counts [76]. Normalization and differential expression analysis were performed with the R/Bioconductor package edgeR v.3.24.3 [77], and statistical significance was assessed applying a general linear model, negative binomial distribution, and quasi-likelihood F test. Genes with a fold change >2 and *p*-value < 0.05 (with a false discovery rate of 5%) were considered differentially expressed.

Genes that were up- and downregulated when comparing Caki-1 and Caki-1-SR cells were analyzed through gene ontology enrichment analysis in Enrichr (http://amp.pharm.mssm.edu/Enrichr, accessed on 3 February 2021). The RNA-Seq data were deposited in GSE172165.

Molecular network and pathway analysis was performed using the MetaCoreTM [78], a web-based tool from Cortellis^TM^ hosted by Clarivate^TM^ (https://clarivate.com/cortellis/, accessed on 3 February 2021).

### 4.9. LC-HRMS/MS Analysis

A total of 1 × 10^6^ cells were seeded in 150 × 15 mm Petri dishes (Corning, 351058) 24 h before the administration of fresh medium containing 0.05% DMSO or 1 µM sunitinib. After 24 h, samples were collected through (i) directly removing 1 mL supernatant and (ii) the addition of 1 mL methanol on top of the cell pellet to extract the cell content.

Chromatographic separation was performed on a Waters Acquity UPLC system interfaced to a Q-Exactive Focus mass spectrometer (Thermo Scientific, Bremen, Germany), using a heated electrospray ionization (HESI-II) source. Thermo Scientific Xcalibur 3.1 software was used for instrument control. The LC conditions were as follows: column, Waters BEH C18 50 × 2.1 mm, 1.7 μm; mobile phase, (A) water with 0.1% formic acid; (B) acetonitrile with 0.1% formic acid; flow rate, 600 μL·min^−1^; injection volume, 2 μL; gradient, a linear gradient of 5–100% B over 7 min and isocratic at 100% B for 1 min. The optimized HESI-II parameters were as follows: source voltage, 3.5 kV (pos); sheath gas flow rate (N2), 55 units; auxiliary gas flow rate, 15 units; spare gas flow rate, 3.0; capillary temperature, 350.00 °C, S-Lens RF Level, 45. The mass analyzer was calibrated using a mixture of caffeine, methionine–arginine–phenylalanine–alanine–acetate (MRFA), sodium dodecyl sulfate, sodium taurocholate, and Ultramark 1621 in an acetonitrile/methanol/water solution containing 1% formic acid by direct injection. The data-dependent MS/MS events were performed on the three most intense ions detected in full scan MS (Top3 experiment). For the metabolites only, MS1 data were considered. The MS/MS isolation window width was 1 Da, and the stepped normalized collision energy (NCE) was set to 15, 30 and 45 units. In data-dependent MS/MS experiments, full scans were acquired at a resolution of 35,000 FWHM (at *m/z* 200) and MS/MS scans at 17,500 FWHM both with an automatically determined maximum injection time. After being acquired in a MS/MS scan, parent ions were placed in a dynamic exclusion list for 2.0 s. Custom exclusion list to remove background ions was used.

The MS data were converted from the RAW (Thermo) standard data format to mzXML format using the MSConvert software, part of the ProteoWizard package [79]. The converted files were treated using the MZMine software suite v. 2.38 [80]. After primary investigation, a targeted list was built for the detection of sunitinib stereoisomers ([M + H]^+^ in positive ion mode 399.2191 *m*/*z* ± 10 ppm) and their *N*-desethyl-forms (371.1878 *m*/*z* ± 10 ppm) (0.1 min retention time tolerance). Peaks containing fewer than 6 data points were rejected. Percent of stereoisomeric excess was calculated as follows:

% stereoisomeric excess (*Z/E*) = ((Peak area *Z*-form)–(Peak area *E*-form))/((Peak area *Z*-form) + (Peak area *E*-form)) × 100

### 4.10. In Vivo Model and 3D Heterotypic Spheroid Cultures from Murine Tumor Tissue

Briefly, female and male Swiss nu/nu mice (NU(Ico)-Foxn1nu) aged 6–8 weeks were obtained from Charles River (Écully, France). For subcutaneous xenografts, mice were inoculated in the left flank with 5 × 106 Caki-1 or Caki-1-SR cells suspended in 100 µL of DMEM medium, supplemented with 1% FCS. One hundred microliters of cell suspension per mouse were injected.

Treatment with 20 mg/kg sunitinib was initiated when palpable tumors had formed (approximately 30 mm^3^) and applied orally for 21 days. After dissection of the tumor, single-cell suspension was prepared in StemPro medium [81]. Cells were kept in culture for 6 days to promote cell aggregation, proliferation, and the natural formation of spheroids and organoid-like constructs. To perform the analysis of treatment response, these aggregates were re-dissociated to obtain a single cell suspension and seeded following the same protocol as 3D cultures from cell lines, distributing 1000 cells into each well.

### 4.11. Statistical Analysis

The data are presented as the mean of multiple independent experiments. Error bars represent the standard error unless otherwise specified. Statistical analysis was performed in Graphpad Prism^®^, version 7.04). Calculation of statistically significant values are given in each figure legend specifically marking *p*-values with *** *p* < 0.001, ** *p* < 0.01 or * *p* < 0.05.

## 5. Patents

P.N.-S. and M.R. are the inventors of WO2021058587 patent on methods of drug com-bination therapy.

## Data Availability

Any underlying research materials related to this manuscript (for example data or models) can be requested by contacting the corresponding author.

## Supplementary Materials

Supplementary materials can be found at https://www.mdpi.com/article/10.3390/ijms22126467/s1.
