# Supplementary material for: Molecular and Functional Analysis of Sunitinib-Resistance Induction in Human Renal Cell Carcinoma Cells
Source: Int J Mol Sci. 2021 Jun 16;22(12):6467. doi: 10.3390/ijms22126467 (PMC8235637; doi:10.3390/ijms22126467)
Supplement: Supplementary file 1 [file ijms-22-06467-s001.zip › ijms-1226882-supplementary.pdf]

Supplementary Materials

Molecular and Functional Analysis of Sunitinib-Resistance Induction in Human Renal Cell Carcinoma Cells

Magdalena Rausch [1,2,3], Adriano Rutz [1,2], Pierre-Marie Allard [1,2], Céline Delucinge-Vivier [4], Mylène Docquier [4,5], Olivier Dormond [6], Jean-Luc Wolfender [1,2], Patrycja Nowak-Sliwinska [1,2,3,*]

[1] School of Pharmaceutical Sciences, University of Geneva, CMU-Rue Michel-Servet 1, CH-1211 Geneva 4, Switzerland; Magdalena.Rausch@unige.ch (M.R.); Adriano.Rutz@unige.ch (A.R.); pierre-marie.allard@unige.ch (P.-M.A.); jean-luc.wolfender@unige.ch (J.-L.W.)
[2] Institute of Pharmaceutical Sciences of Western Switzerland, University of Geneva, CMU-Rue Michel-Servet 1, CH-1211 Geneva 4, Switzerland
[3] Translational Research Center in Oncohaematology, 1205 Geneva, Switzerland
[4] iGE3 Genomics Platform, University of Geneva, Geneva, Switzerland; Celine.Delucinge@unige.ch (C.D.-V.); Mylene.Docquier@unige.ch (M.D.)
[5] Department of Genetics and Evolution, University of Geneva, 1205 Geneva, Switzerland
[6] Department of Visceral Surgery, Lausanne University Hospital and University of Lausanne, 1015 Lausanne, Switzerland; olivier.dormond@chuv.ch
* Correspondence: Patrycja.Nowak-Sliwinska@unige.ch; Tel.: +41-22-379-3352

Supplementary Methods

Cells

Human immortalized mascrovascular endothelial cells (ECRF24) and human fibroblast NHDFα cells were kindly provided by Angiogenesis Laboratory, UMC Amsterdam and Pharmacognosy Group UNIGE Geneva, respectively. ECRF24 cells were maintained in a 50:50 mixture of DMEM and RPMI in a flask pre-coated with 0.2% gelatin (Sigma Aldrich, Buchs, Switzerland, G1393-100ML). All media were supplemented with 10% fetal bovine serum (Biowest, Nuaillé, France, S1810-500) and 1% penicillin/streptomycin (Bioconcept, Basel, Switzerland, 4-01F00-H). NHDFα cells were cultured in a specified culture medium for fibroblasts including a supplement kit (Vitaris, Baar, Switzerland, C-23110-PRO).

Therapeutically Guided Multidrug Optimization

The optimized drug combination consisting of AZD4547, osimertinib, AZD8055, and pictilisib was established to target selectively Caki-1-SR (clone 1) cells using the phenotypic technique described previously [1]. Briefly, analyzing the inhibition of the cell metabolic activity was used to determine the efficacy of each drug combination RCC and non-cancerous cell lines. The drug combination was defined through *in vitro* and *in silico* validation applying the second-order step-wise linear regression models described, as previously described [2]. Drugs were further selected after three sequential search rounds and interpreting the treatment efficacy and its statistical measures, which define the accuracy and reliability of the regression models for each iteration, with an ANOVA lack of fit test confirming the selection of a relevant model structure. Finally, dose-optimization was performed for the cell-type-specific four-drug combination.

Supplementary Figures

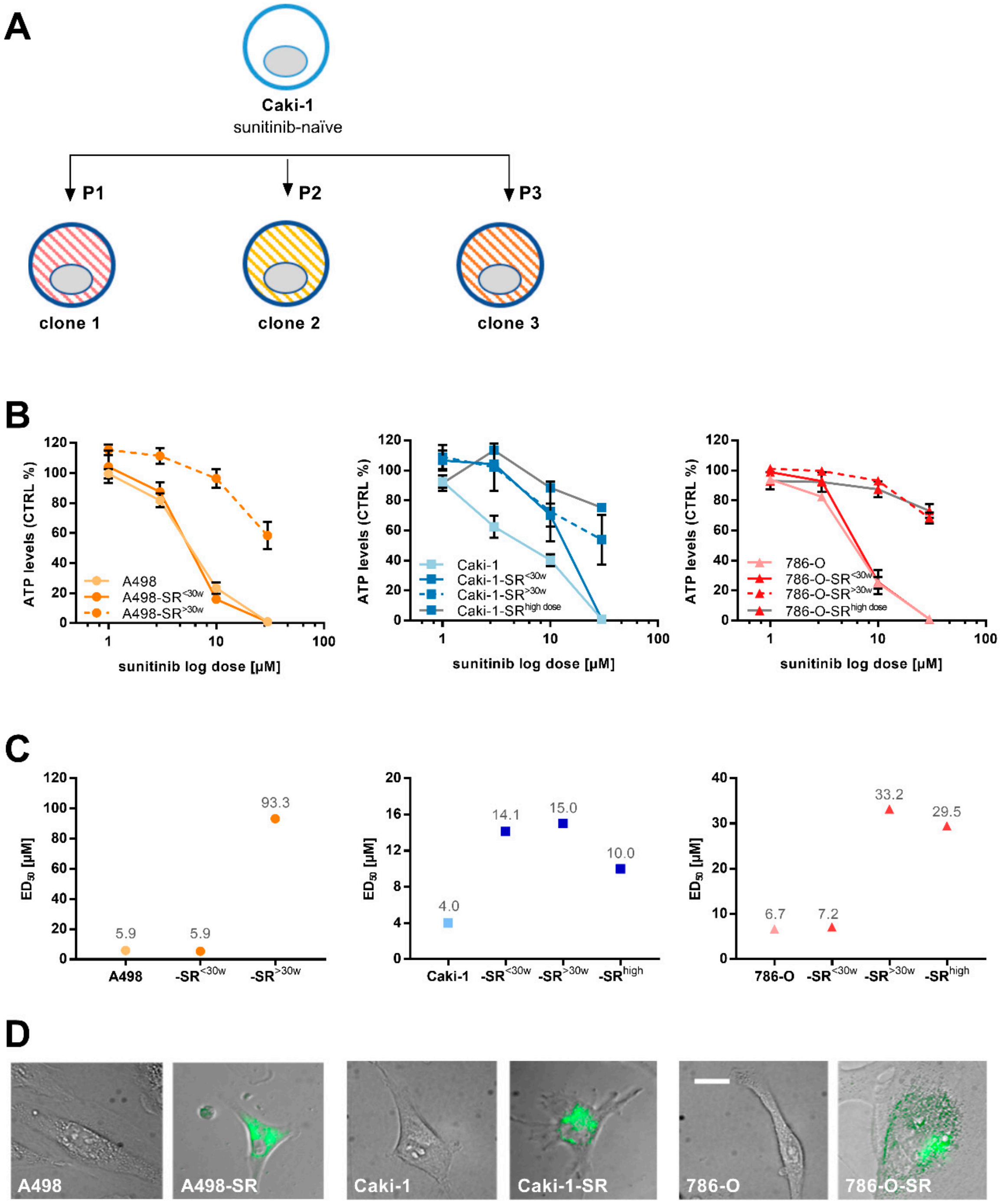

Supplementary Figure S1. Phenotypic analysis of sunitinib-naïve and sunitinib-resistant ccRCC cells. **A**. Schematic representation of the induction and maintenance of resistance to sunitinib following three experimental protocols (P1-P3) to obtain three different clones. P1 = increasing doses of sunitinib (1-30 µM); P2 = continued treatment with 1 µM of sunitinib; P3 = single treatment with 10 µM of sunitinib. **B**. Growth curves of human ccRCC cell lines A498, Caki-1 and 786-O in response to increasing doses of sunitinib measured as the ATP levels in the cells. Growth curves are presented in comparison to the equivalent cell lines chronically treated with 1 µM sunitinib for less than 30 weeks (30w), over 30 weeks, or once - with a high dose of 10 µM sunitinib. Error bars represent the SD. **C**. Calculation of the ED_{50} for each condition and each cell line using a non-linear fit of log(inhibitor) *versus* resoponse (four-parameter variable slope). **D**. Representative bright-field images of sunitinib-naïve and -resistant ccRCC cells demonstrating the accumulation of sunitinib (green) in the lysosomes. Scale bar = 10 µm.

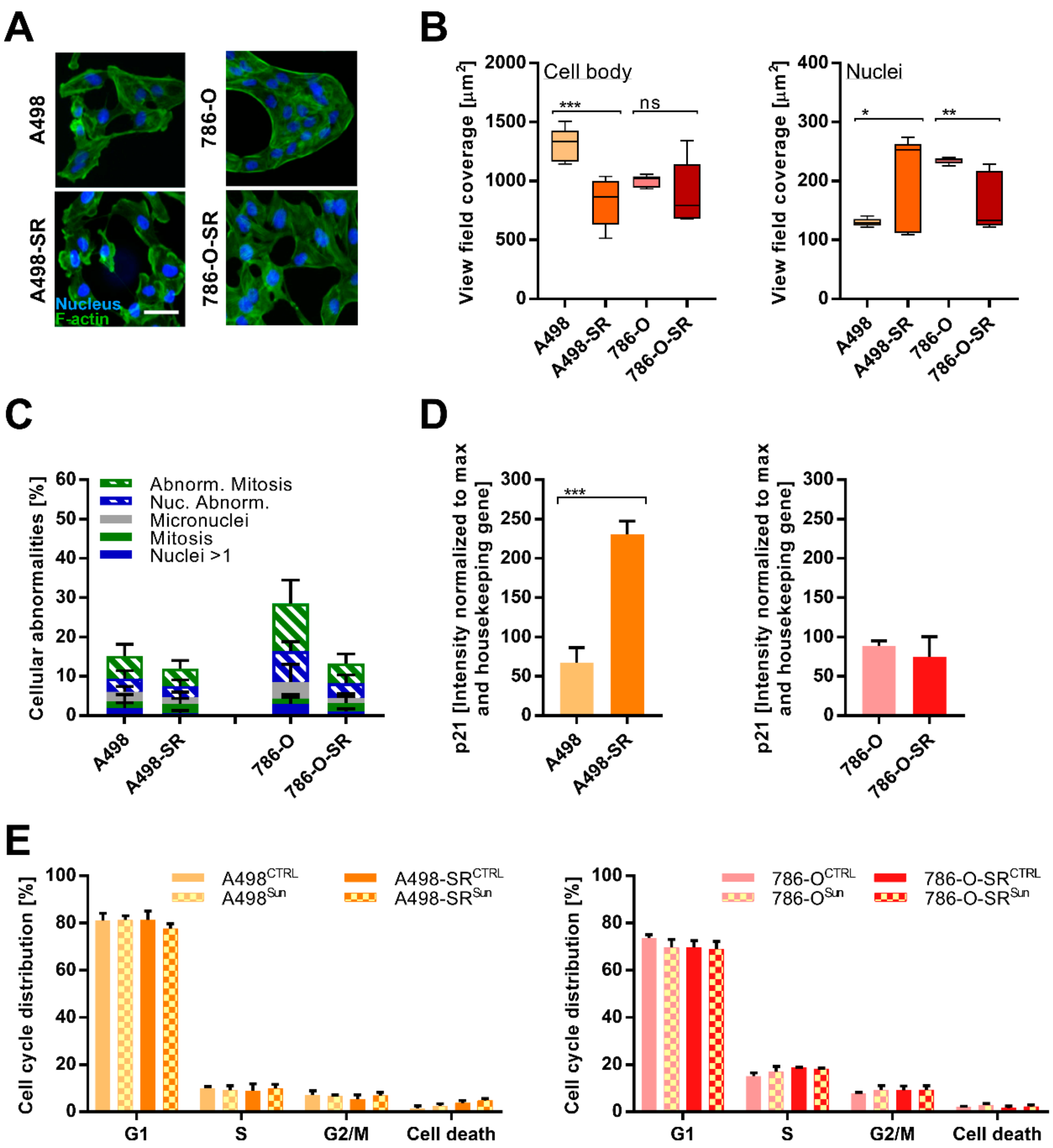

Supplementary Figure S2. Variance between sunitinib-naïve and sunitinib-resistant ccRCC cells after 30 weeks of chronic treatment. **A**. Fluorescent images of A498(-SR) and 786-O(-SR) cells showing the cell body (F-actin; green) and the nucleus (Dapi; blue). Scale bar = 20 µm. **B**. The size of the cell body (left graph) and the nucleus (right graph) of A498(-SR) and 786-O(-SR) cells presented as the area covered in the view field of n = 12 independent images. **C**. Cellular abnormalities measured in A498(-SR) and 786-O(-SR) cells. **D**. The expression of p21 in A498(-SR) (left graph) and 786-O(-SR) cells (right graph). The protein levels have been detected through western blot and quantified normalizing the intensity signal to the maximal intensity of protein detected as well as the house keeping gene 1. **E**. Cell cycle distribution of A498(-SR) (left graph) and 786-O(-SR) (right graph) cells in absence (CTRL) and presence of 1 µM sunitinib (Sun). Cells were localized in the mitotic phases of G1, S, and G2/M. A small proportion (< 5%) was found to undergo cell death. Error bars represent the SD. Significance was calculated using a student's t-test. $p^* < 0.05$, $p^{**} < 0.01$, $p^{***} < 0.001$.

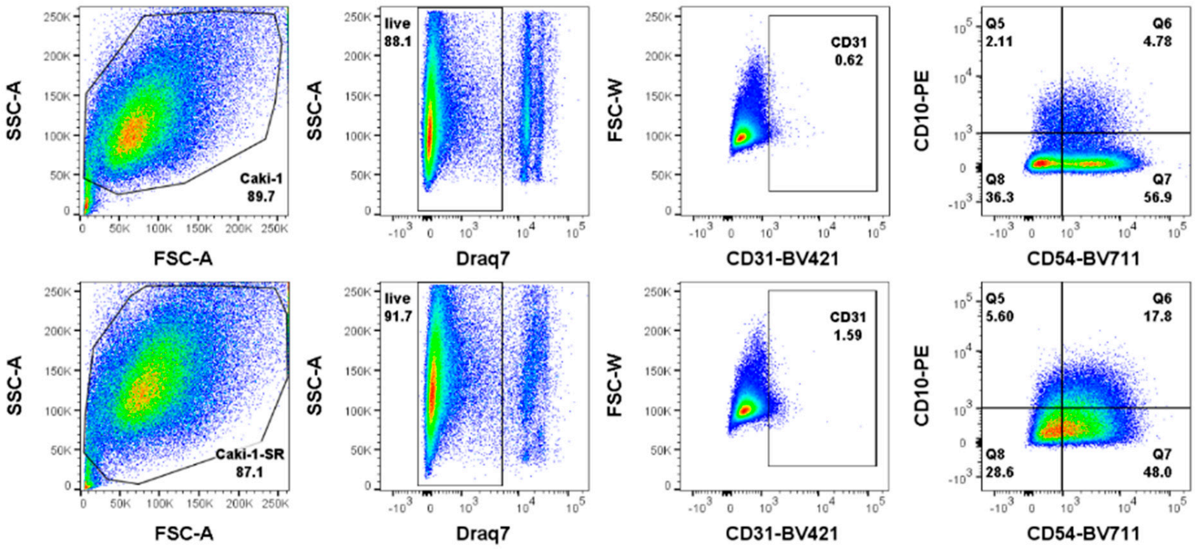

Supplementary Figure S3. Flow cytometry analysis of Caki-1 and Caki-1-SR cells characterizing the expression of distinct cell surface proteins. Caki-1 (top) and Caki-1-SR cells (bottom) were analyzed for their size and granularity through the forward and sideward scatter signal (FSC/ SSC). The viability of the cells was measured through viability staining with Draq7. Focusing on the viable population (live) the surface protein expression of CD31 (platelet endothelial cell adhesion molecule, PECAM-1). Zooming into the CD31 positive population, cells were characterized for the expression of CD10 (cell membrane metallopeptidase) and CD54 (intercellular adhesion molecule 1, ICAM-1). Q5: CD10+CD54- cells; Q6: CD10+CD54+ cells; Q7: CD10-CD54+ cells; Q8 CD10-CD54- cells.

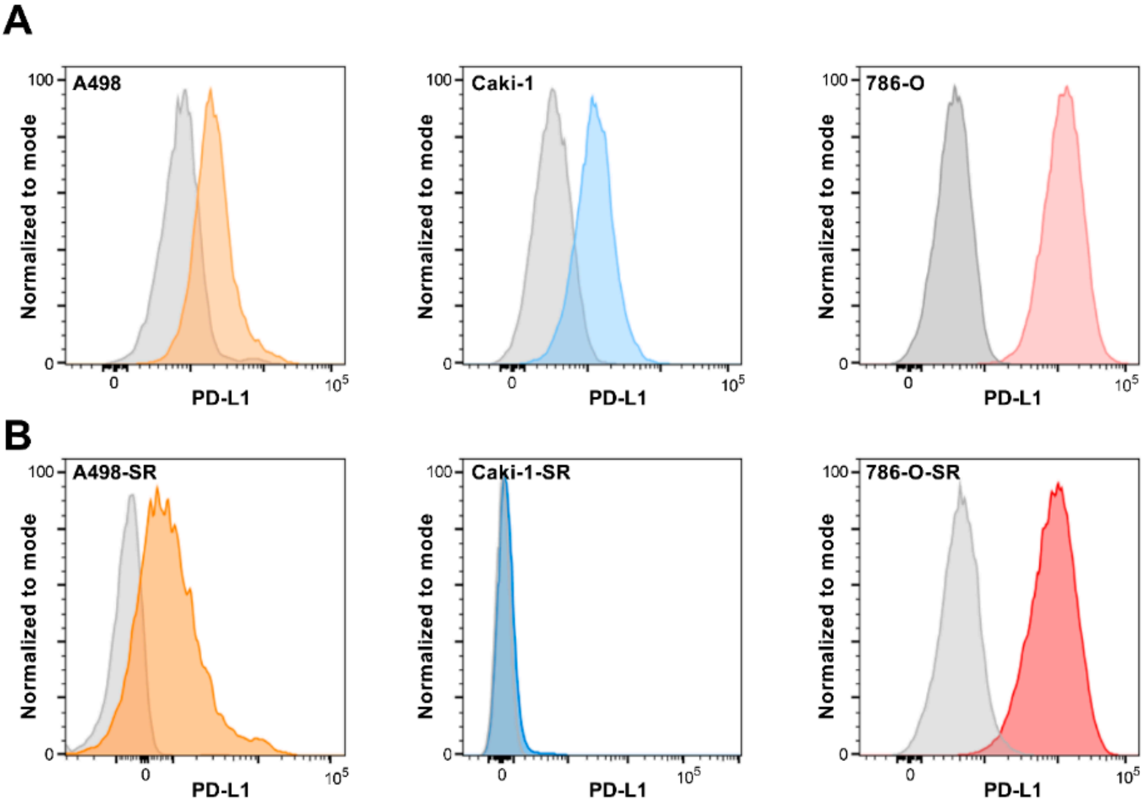

Supplementary Figure S4. PD-L1 expression of sunitinib-naïve and sunitinib-resistant ccRCC cell lines. Histograms showing the expression of PD-L1 measured through flow cytometry analysis on the surface of sunitinib naïve (**A**) and resistant (**B**) ccRCC cell lines. The expression of PD-L1 is demonstrated as the fluorescence signal intensity (colored peaks) in comparison to unstained cells (gray peak).

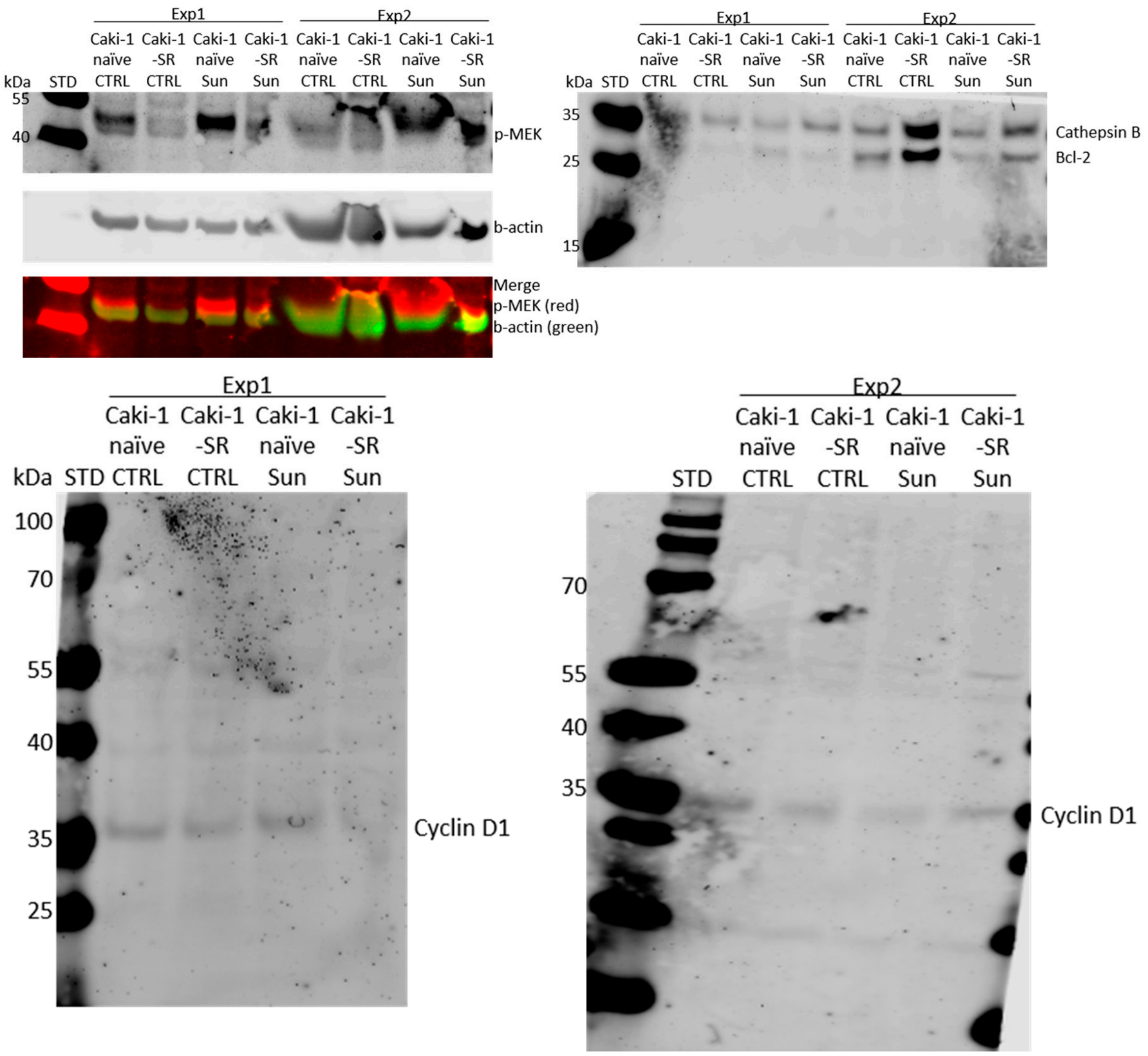

Supplementary Figure S5. Full blots of protein expression in sunitinib-naïve and sunitinib-resistant ccRCC cell lines. Protein expression analysis in Caki-1 and Caki-1-SR cells untreated (CTRL) or treated for 72 hours with 1 µM sunitinib (Sun). Through western blot experiments the protein level of phosphorylated (p-) mitogen activated kinase kinase (MEK; 45 kDa), cathepsin B (44 kDa), Bcl-2 (26 kDa), cyclin D1 (36 kDa) and the housekeeping gene β-actin (45 kDa). The original blots show the comparison of 2 independet experiments (Exp1 and Exp2, N = 2).

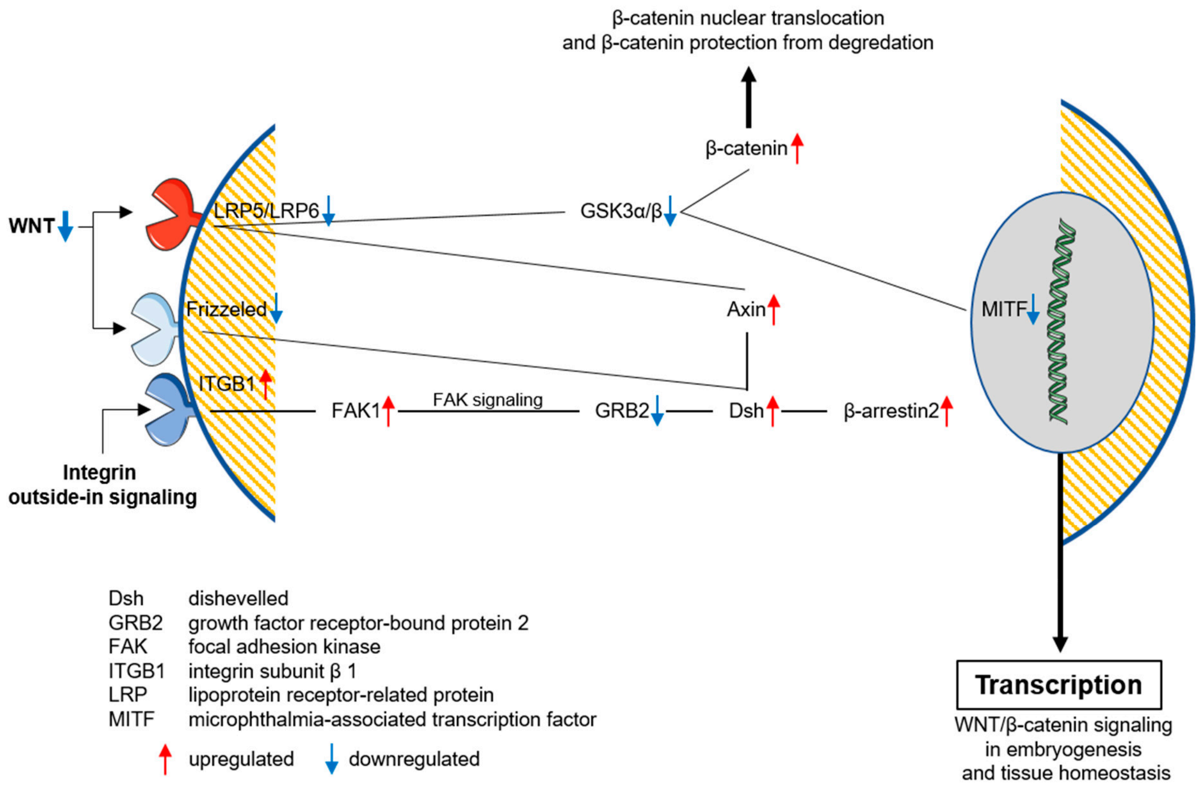

Supplementary Figure S6. Pathway analysis highlighting the difference in activation of the WNT/ β-catenin pathway comparing Caki-1 and Caki-1-SR cells. Induced through integrin outside-in signaling, the MetaCoreTM pathway analysis revealed a strong downregulation of WNT and the transcription factor β-catenin, which causes a reduced singaling via the intercalated signaling pathway. This down-regulation has an ultimate impact on the transcription of proteins participating in embryogenesis and tissue homeostasis. The map demonstrates the connections between the single components in the WNT/ β-catetnin signaling.

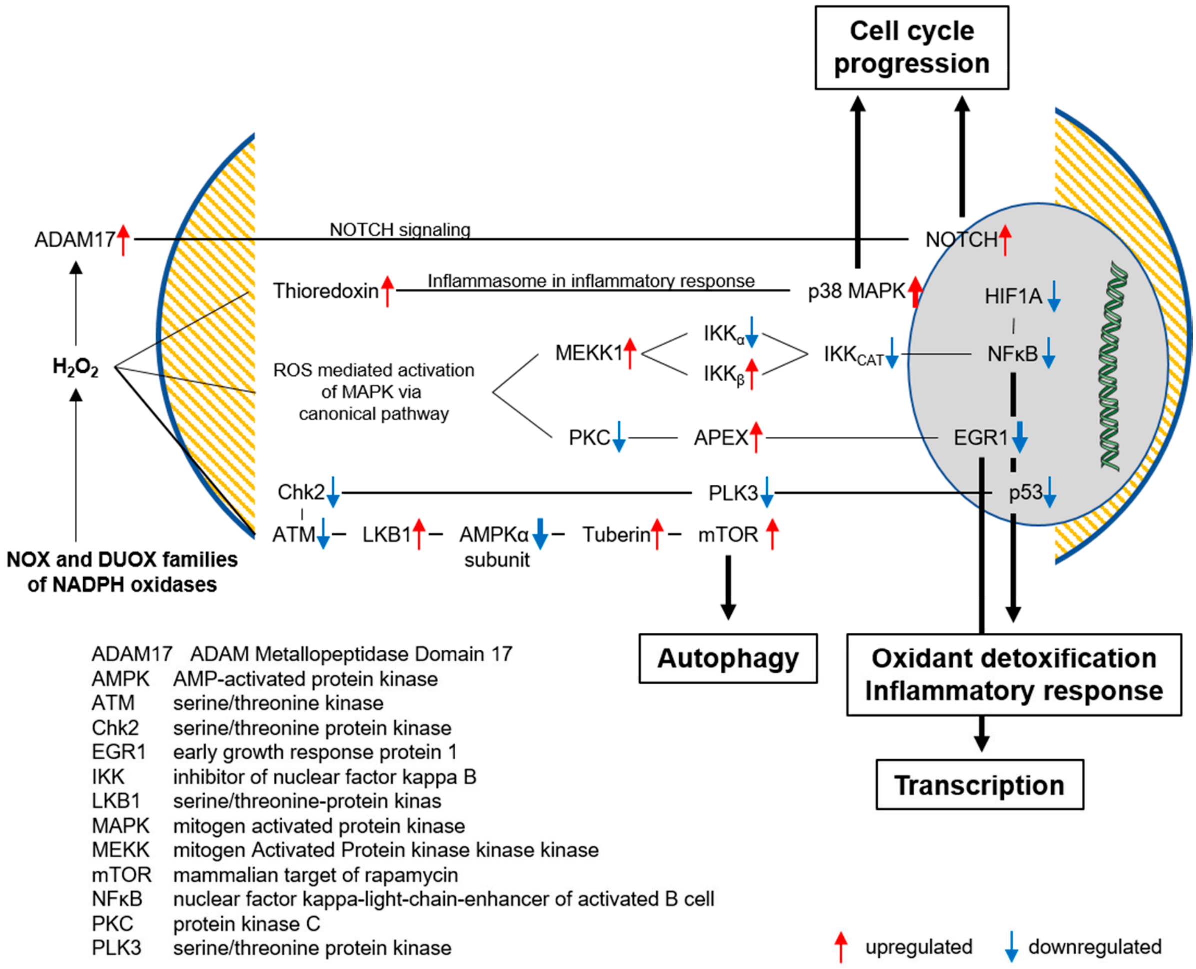

Supplementary Figure S7. Pathway analysis highlighting the difference in response to reactive oxygen species accumulation comparing Caki-1 and Caki-1-SR cells. Map of the molecular pathway analysis performed with MetaCoreTM demonstrating the consequences of Adam17 activation regulating Notch3 processing related to altered response to reactive oxygen species. From the top to the bottom the signal transduction is presented highlighting the cellular responses that depend on the alterations within the cascade.

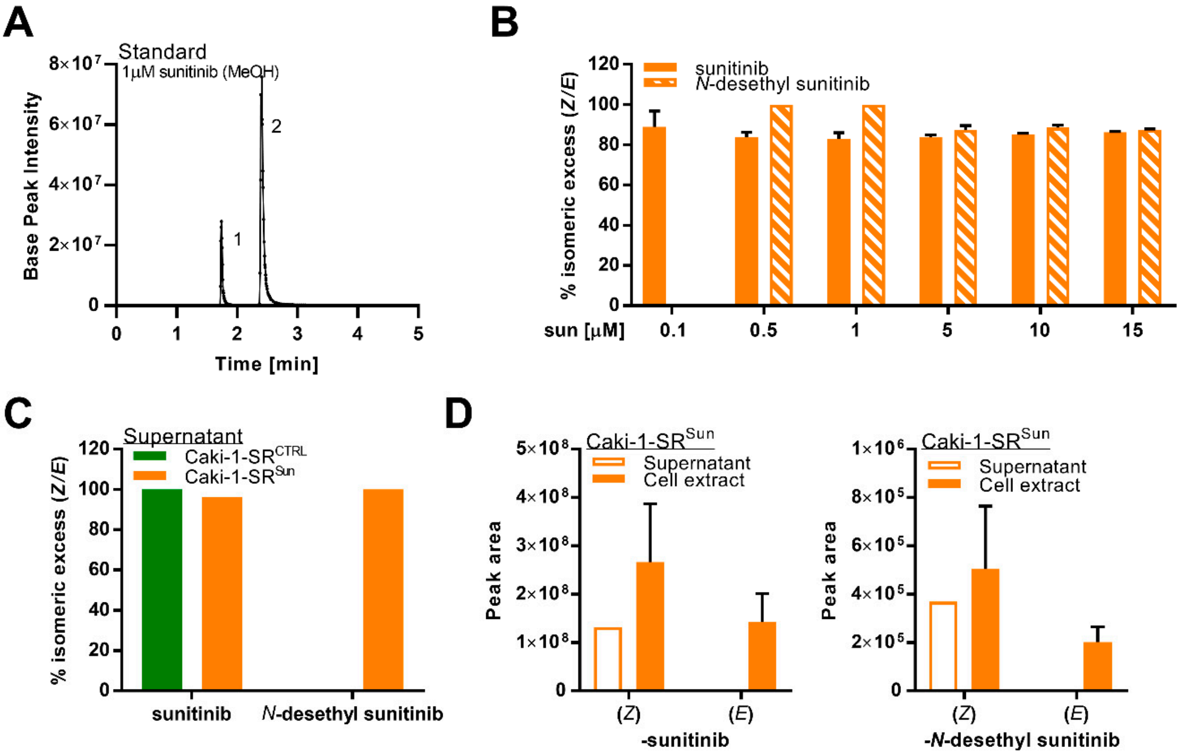

Supplementary Figure S8. LC-HRMS-MS analysis of sunitinib and related compounds. **A**. Extracted Ion Chromatogram (XIC at 399.22 m/z) of the sunitinib standard. The (*E*)- and (*Z*)- stereoisomers (1 and 2 respectively) are presented. **B**. Representation of the percentage of stereoisomeric excess of (*Z*)-form over (*E*)-form in the supernatant of Caki-1-SR cells treated with increasing concentrations of sunitinib. The percentage of stereoisomeric excess is independent of the treatment concentration and similar between sunitinib and its metabolite, *N*-desethyl sunitinib. **C**. Bar graphs demonstrating the % isomeric excess (Z/E) of the sunitinib isomers and their N-desethyl metabolites in the supernatant (left graph; N = 1) of Caki-1-SR cells cultured for 24 hours in culture medium (CTRL) or in culture medium supplemented with 1 µM sunitinib. **D**. Bar graphs showing the peak area of sunitinib (left graph) and *N*-desethyl sunitinib (right graph) measured in the supernatant and the cell extract of Caki-1-SR cells treated for 24 hours with 1 µM sunitinib. In the supernatant only the (*Z*)-form was detected. In the cell extract both isomer forms were detected.

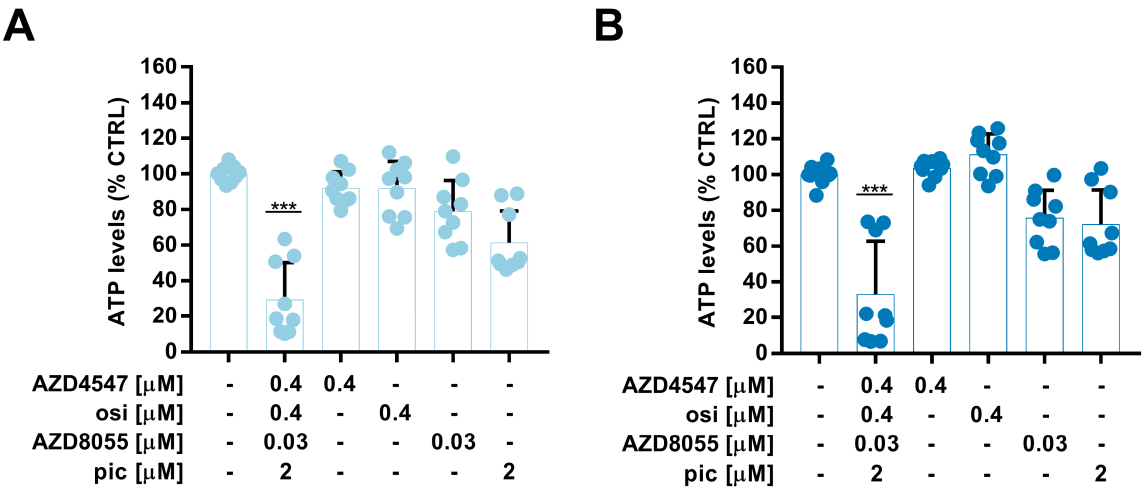

Supplementary Figure S9. Cell metabolic activity measurements of Caki-1 and Caki-1-SR-based heterotypic 3D co-cultures after 72 hours of treatment with the ODC. ATP levels of Caki-1-based (A) and Caki-1-SR-based (B) heterotypic 3D co-cultures measured after 72 hours of treatment with the ODC and its monotherapies. Error bars represent the SD. Statistical significance calculated using a two-way ANOVA analysis. N = 3; p*** < 0.001.

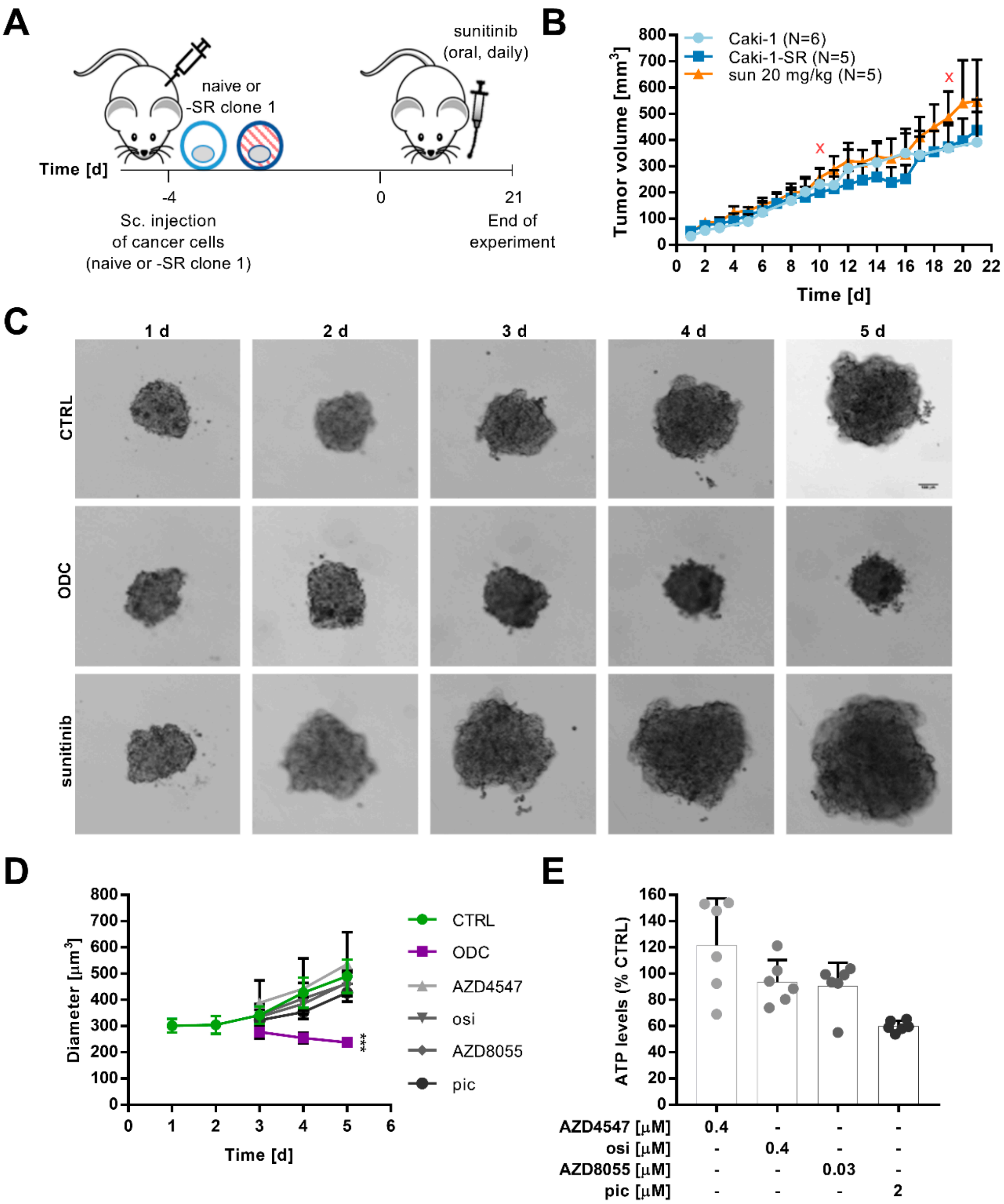

Supplementary Figure S10. Preparation of Caki-1-SR-based heterotypic ex vivo organoid-like cultures for the testing of a drug combination and its single drugs. A. Scheme of the experimental procedure conducting an in vivo study on the tumor growth of Caki-1 sunitinib-naïve and –resistant cells. $1x10^6$ Caki-1 or Caki-1-SR clone 1 cells were injected in the subcutaneous flank of Swiss nude mice. After a palpable tumor formed, sunitinib was given orally to N = 5 mice at a daily dose of 20 mg/kg for 21 days. B. Growth curves of Caki-1, Caki-1-SR and sunitinib treated Caki-1-SR tumors monitored for 21 days. Two mice showed severe side effects in response to sunitinib treatment and had to be sacrificed (red cross). C. Bright-field images of Caki-1-SR-based heterotypic ex vivo organoid-like cultures representing the development over 5 days (1-5d). The organoid-like cultures were cultured in culture medium (CTRL) or culture medium supplemented with either the multi-drug combination ODC or 1 µM sunitinib. Scale bar = 100 µm. D. Size measurements of the organoid-like cultures in response to the ODC and its monotherapies. E. Bar graphs showing the ATP levels of the Caki-1-SR-based organoid-like cultures after 72 hours of treatment with the monotherapies at the given doses. Error bars represent the SD.

Supplementary Tables

Supplementary Table S1. Transcripts related to lysosomal trafficking and autophagy.

Gene	Full Name	Fold Change	Raw *p*-Value	*p*-Value with FDR	Regulation
ATG101	autophagy related 101	1.11	0.0439	0.1065	upregulated
**ATG13	autophagy related 13	1.25	0.0008	0.0057	upregulated
*ATG16L1	autophagy related 16 like 1	1.20	0.0121	0.0410	upregulated
ATG16L2	autophagy related 16 like 2	1.32	0.1818	0.3055	upregulated
ATG2B	autophagy related 2B	1.00	0.9925	0.9956	upregulated
ATG3	autophagy related 3	1.03	0.5253	0.6548	upregulated
ATG4A	autophagy related 4A cysteine peptidase	1.06	0.5300	0.6587	upregulated
*ATG4B	autophagy related 4B cysteine peptidase	1.22	0.0107	0.0374	upregulated
ATG4D	autophagy related 4D cysteine peptidase	1.18	0.1417	0.2534	upregulated
**ATG7	autophagy related 7	1.33	0.0008	0.0058	upregulated
ATG9A	autophagy related 9A	1.04	0.4314	0.5701	upregulated
**LAMP1	lysosomal associated membrane protein 1	1.26	0.0005	0.0042	upregulated
LAMTOR2	late endosomal/lysosomal adaptor, MAPK and mTOR activator 2	1.08	0.3184	0.4609	upregulated
*LAMTOR4	late endosomal/lysosomal adaptor, MAPK and mTOR activator 4	1.40	0.0096	0.0343	upregulated
MAP1LC3B2	microtubule associated protein 1 light chain 3 beta 2	1.43	0.3605	0.5023	upregulated
ATG10	autophagy related 10	-1.11	0.2569	0.3942	downregulated
ATG12	autophagy related 12	-1.08	0.2324	0.3667	downregulated
ATG14	autophagy related 14	-1.19	0.0637	0.1407	downregulated
ATG2A	autophagy related 2A	-1.05	0.4493	0.5866	downregulated
**ATG4C	autophagy related 4C cysteine peptidase	-1.51	0.0005	0.0039	downregulated
ATG5	autophagy related 5	-1.13	0.0774	0.1627	downregulated
LAMP2	lysosomal associated membrane protein 2	-1.15	0.0201	0.0597	downregulated
LAMTOR1	late endosomal/lysosomal adaptor, MAPK and mTOR activator 1	-1.04	0.4221	0.5615	downregulated
LAMTOR3	late endosomal/lysosomal adaptor, MAPK and mTOR activator 3	-1.05	0.4638	0.6000	downregulated
LAMTOR5	late endosomal/lysosomal adaptor, MAPK and mTOR activator 5	-1.17	0.0262	0.0730	downregulated
MAP1LC3B	microtubule associated protein 1 light chain 3 beta	-1.12	0.0551	0.1261	downregulated

** and * = significance ($p^* < 0.05$, $p^{**} < 0.01$).

Supplementary Table S2. Significantly dysregulated transcripts related to cellular adhesion processes.

Gene	Full Name	Fold Change	*p*-Value	*p*-Value with FDR	Regulation
ADGRA3	adhesion G protein-coupled receptor A3	1.20	0.0057	0.0233	upregulated
ANXA8	annexin A8	10.08	0.0008	0.0057	upregulated
ANXA11	annexin A11	1.18	0.0027	0.0137	upregulated
ECM1	extracellular matrix protein 1	2.14	0.0042	0.0187	upregulated
GDF15	growth differentiation factor 15	4.81	0.0000	0.0001	upregulated
ILK	integrin linked kinase	1.29	0.0003	0.0027	upregulated
MFAP5	microfibril associated protein 5	2.60	0.0008	0.0058	upregulated
MMP1	matrix metallopeptidase 1	3.07	0.0069	0.0269	upregulated
MMP13	matrix metallopeptidase 13	3.03	0.0195	0.0584	upregulated
MMP15	matrix metallopeptidase 15	2.07	0.0031	0.0149	upregulated
NDNF	neuron derived neurotrophic factor	2.66	0.0031	0.0152	upregulated
PTX3	pentraxin 3	2.31	0.0000	0.0002	upregulated
SHH	sonic hedgehog signaling molecule	4.81	0.0002	0.0020	upregulated
THBS1	thrombospondin 1	1.58	0.0000	0.0005	upregulated
THBS4	thrombospondin 4	1.97	0.0230	0.0663	upregulated
ABI3BP	ABI family member 3 binding protein	-3.46	0.0107	0.0375	downregulated
ADGRB3	adhesion G protein-coupled receptor B3	-1.35	0.0221	0.0644	downregulated
ADGRG1	adhesion G protein-coupled receptor G1	-1.49	0.0000	0.0004	downregulated
ADGRG6	adhesion G protein-coupled receptor G6	-1.31	0.0009	0.0063	downregulated
ADGRL1	adhesion G protein-coupled receptor L1	-1.43	0.0002	0.0023	downregulated
BCAM	basal cell adhesion molecule (Lutheran blood group)	-4.05	0.0001	0.0015	downregulated
BGN	biglycan	-5.15	0.0037	0.0170	downregulated
CFP	complement factor properdin	-2.13	0.0036	0.0167	downregulated
COL11A1	collagen type XI alpha 1 chain	-79.02	0.0000	0.0000	downregulated
COL12A1	collagen type XII alpha 1 chain	-2.12	0.0000	0.0005	downregulated
COL14A1	collagen type XIV alpha 1 chain	-1.83	0.0001	0.0012	downregulated
COL17A1	collagen type XVII alpha 1 chain	-1.40	0.0338	0.0882	downregulated
COL1A1	collagen type I alpha 1 chain	-3.24	0.0000	0.0000	downregulated
COL24A1	collagen type XXIV alpha 1 chain	-6.47	0.0003	0.0027	downregulated
COL26A1	collagen type XXVI alpha 1 chain	-6.53	0.0000	0.0004	downregulated
COL4A1	collagen type IV alpha 1 chain	-1.54	0.0000	0.0001	downregulated
COL4A2	collagen type IV alpha 2 chain	-1.28	0.0037	0.0170	downregulated
COL4A4	collagen type IV alpha 4 chain	-3.37	0.0005	0.0039	downregulated
COL4A5	collagen type IV alpha 5 chain	-1.50	0.0001	0.0010	downregulated
COL4A6	collagen type IV alpha 6 chain	-2.10	0.0001	0.0011	downregulated
COL5A2	collagen type V alpha 2 chain	-10.22	0.0000	0.0000	downregulated
COL6A3	collagen type VI alpha 3 chain	-12.47	0.0000	0.0000	downregulated
COL7A1	collagen type VII alpha 1 chain	-1.33	0.0262	0.0730	downregulated
COL8A1	collagen type VIII alpha 1 chain	-2.16	0.0000	0.0000	downregulated
FLG	filaggrin	-2.12	0.0000	0.0000	downregulated
FN1	fibronectin 1	-3.23	0.0000	0.0004	downregulated
HAPLN1	hyaluronan and proteoglycan link protein 1	-2.65	0.0038	0.0173	downregulated
INHBE	inhibin subunit beta E	-2.35	0.0034	0.0160	downregulated
L1CAM	L1 cell adhesion molecule	-2.84	0.0000	0.0000	downregulated
LAMA1	laminin subunit alpha 1	-1.87	0.0009	0.0060	downregulated
LAMA2	laminin subunit alpha 2	-1.66	0.0129	0.0431	downregulated
LAMA3	laminin subunit alpha 3	-6.63	0.0000	0.0000	downregulated
LAMA4	laminin subunit alpha 4	-41.76	0.0000	0.0001	downregulated
LAMB3	laminin subunit beta 3	-1.77	0.0000	0.0001	downregulated
LAMC2	laminin subunit gamma 2	-1.72	0.0010	0.0068	downregulated
MMP19	matrix metallopeptidase 19	-2.60	0.0151	0.0483	downregulated
SERPINA1	serpin family A member 1	-2.25	0.0009	0.0062	downregulated
THBS3	thrombospondin 3	-2.16	0.0128	0.0428	downregulated

Supplementary Table S3. RNA sequencing data showing the expression of VHL, c-MET, VEGF, mTOR and PDGF(R) as well as related transcripts.

Gene	Description	Fold Change	*p*-Value	*p*-Value with FDR	Regulation
VBP1	VHL binding protein 1	−1.15	0.1092	0.2096	downregulated
VHL	von Hippel-Lindau tumor suppressor	−1.10	0.0958	0.1901	downregulated
MET	MET proto-oncogene, receptor tyrosine kinase	1.13	0.0376	0.0953	upregulated
VEGFA	vascular endothelial growth factor A	−1.22	0.0312	0.0830	downregulated
VEGFB	vascular endothelial growth factor B	1.08	0.2860	0.4263	upregulated
VEGFC	vascular endothelial growth factor C	1.14	0.0645	0.1421	upregulated
MTOR	mechanistic target of rapamycin kinase	1.23	0.0013	0.0080	upregulated
PDGFA	platelet derived growth factor subunit A	1.64	0.0012	0.0077	upregulated
PDGFB	platelet derived growth factor subunit B	2.41	0.0000	0.0004	upregulated
PDGFC	platelet derived growth factor C	−1.19	0.0108	0.0376	downregulated
PDGFD	platelet derived growth factor D	1.31	0.0178	0.0546	upregulated
PDGFRB	platelet derived growth factor receptor beta	−2.14	0.0004	0.0035	downregulated
PDGFRL	platelet derived growth factor receptor like	4.25	0.0044	0.0192	upregulated

Supplementary Table S4. Small molecule-based compounds used in this study.

Compound	Cellular Target	Development	ODC [μM]
AZD4547	FGFR1/2/3, FGFR4, VEGFR2	Phase IIa	0.4
AZD8055	mTORC1/2	Phase I/II	0.03
osimertinib	EGFR	Approved	0.4
Pictilisib (GDC-0941)	PI3Kα/δ	Phase II	2.0

Supplementary Table S5. Fluorophore conjugated antibodies for flow cytometry analisis.

Marker	Fluorochrome	Antibody Type	Dilution	Provider	Reference
CD10	PE	Mouse a-human IgG1	1:500	BD Bioscience	557143
CD31	BV421	Mouse a-human IgG1	1:1000	BD Bioscience	564089
CD54 (ICAM-1)	BV711	Mouse a-human IgG3	1:1000	BD Bioscience	564078
CD274 (PD-L1)	APC	Mouse a-human IgG1	1:1000	Biolegend	329708

Supplementary Table S6. Unconjugated primary antibodies for western blot analysis.

Protein	Molecular Weight [kDa]	Specification	Species	Dilution	Provider	Reference
β-actin	45	clone AC-74	Mouse	1:4000	Sigma Aldrich	A2228
Bcl-2	26	124	Mouse	1:4000	CellSignaling	15071S
Cathepsin B	44, 27, 24	D1C7Y	Rabbit	1:4000	CellSignaling	31718S
Cyclin D1	36	92G2	Rabbit	1:4000	CellSignaling	2978S
phospho-MEK1/2	45	Ser217/221	Rabbit	1:4000	CellSignaling	9121S
p21	21	Waf1/Cip1 (12D1)	Rabbit	1:4000	CellSignaling	2947S

kDa = kilodalton; phospho- = phosphorylated.

Supplementary References

1. Rausch, M.; Weiss, A.; Achkhanian, J.; Rotari, A.; Nowak-Sliwinska, P. Identification of low-dose multidrug combinations for sunitinib-naive and pre-treated renal cell carcinoma. *Br. J. Cancer* **2020**, *123*, 556–567, doi:10.1038/s41416-020-0890-y.
2. Weiss, A.; Berndsen, R.H.; Ding, X.; Ho, C.M.; Dyson, P.J.; Van Den Bergh, H.; Griffioen, A.W.; Nowak-Sliwinska, P. . A streamlined search technology for identification of synergistic drug combinations. *Sci Rep.* 2015, *5*, 14508, doi:10.1038/srep14508.
